# Tethered release of the pseudorabies virus deubiquitinase from the capsid promotes enzymatic activity

**DOI:** 10.1128/jvi.01517-24

**Published:** 2024-12-05

**Authors:** Sarah E. Antinone, John S. Miller, Nicholas J. Huffmaster, Gary E. Pickard, Gregory A. Smith

**Affiliations:** 1 Department of Microbiology–Immunology, Northwestern University Feinberg School of Medicine 547641 , Chicago, Illinois, USA; 2 School of Veterinary Medicine and Biomedical Sciences, University of Nebraska—Lincoln 684783 , Lincoln, Nebraska, USA; University of Virginia, Charlottesville, Virginia, USA

**Keywords:** alphaherpesvirus, pseudorabies virus, PRV, large tegument protein, virion structure, pUL36, deubiquitinase, DUB, cysteine, capsid

## Abstract

Herpesviruses carry an assortment of proteins in the interstitial space between the capsid and membrane envelope, collectively referred to as the tegument. Upon virion fusion with a cell, envelope integrity is disrupted, and many tegument constituents disperse into the cytosol to carry out individual effector functions, while others direct transport of the capsid to the nucleus. To gain insight into the tegument dynamics that occur with disruption of envelope integrity, we used a combination of single-particle fluorescence and biochemical approaches that leveraged the previously established use of n-ethylmaleimide to inhibit virion dynamics. We document that the large tegument protein (pUL36), which is stably bound to the capsid surface at its C-terminus, is also conditionally bound to the capsid via its N-terminal deubiquitinase (DUB) domain. The DUB is released, while remaining tethered to the capsid by the pUL36 C-terminus, by a mechanism dependent on reactive cysteines. Mutation of these cysteines locks the DUB in a capsid bound state and suppresses enzymatic activity.

**IMPORTANCE:**

Neuroinvasive alphaherpesviruses, such as herpes simplex virus and pseudorabies virus, cause a broad range of diseases in humans and other animals. Novel strategies to interfere with the virion structural rearrangements required for infectivity could prove valuable to treat infections, yet critical aspects of the virion architecture and its metastability remain poorly defined. In this study, we document that the pUL36 tegument protein exhibits conditional capsid binding in its N-terminal deubiquitinase domain that regulates enzymatic activity during infection.

## INTRODUCTION

A distinguishing feature of herpesviruses is the large assortment of proteins that are incorporated between the capsid shell and the lipid bilayer envelope of their virions. This protein matrix is referred to as the herpesvirus tegument. Tegument proteins play structural roles in assembly of virions by connecting the capsid and envelope through a series of protein–protein interactions. Upon virus entry, these structural components are once again exposed to the cytosol and disperse to effect changes to cellular homeostasis on a rapid time scale that precedes viral gene expression (1). Tegument proteins that are effectors of capsid intracellular trafficking remain capsid-bound upon infection, whereas the majority of tegument proteins dissociate from the incoming viral particle and perform a diverse set of functions to prime the cell toward a permissive environment that is supportive of viral infection (2–18). The metastability of the tegument is, therefore, essential to its overall function: promoting viral egress by assembling into newly forming virions during late infection and performing effector functions that promote viral ingress and prime infection following entry into the next cell.

The tegument is commonly referred to as having two layers: an inner layer that uses the capsid as a scaffold for its assembly and an outer layer that is initially associated with the nascent viral envelope. The two layers are thought to come together when capsids become enveloped in the cytosol. While this depicts a model in which tegument proteins agglutinate to form the virion particle, several lines of investigation indicate that the tegument is dynamic and, when mature, is poised to undergo additional architectural alterations that promote infection (19–24).

The large inner tegument protein, pUL36 (aka VP1/2), forms the only established link between the tegument and capsid in the alphaherpesvirinae by interfacing with the pUL25 capsid protein via its C-terminal capsid-binding domain (CBD) (25–30). Upon entering a cell, pUL36 remains capsid bound and promotes trafficking of the capsid along microtubules to nuclear pores where it then promotes injection of the viral DNA into the nucleus (2, 3, 7, 8, 31–34). Despite these defined functions, a truncation mutant of pUL36 that lacks the C-terminal CBD remains competent to assemble virions but, upon entering cells, fails to retain pUL36 on the capsid, resulting in an abortive infection (35). This finding indicates that, in addition to the C-terminal CBD, pUL36 has other domains that promote its packaging into the tegument.

We report that the N-terminal deubiquitinase (DUB) domain of pseudorabies virus (PRV) pUL36 uses cysteine chemistry to dynamically interact with the capsid surface. The enzymatic activity of the DUB is restrained in the capsid bound state. Upon disruption of the virion envelope, the N-terminus is released from the capsid surface but remains tethered by the C-terminal CBD. Data are provided indicating that the DUB is enzymatically active in the extended configuration. The findings demonstrate that the pUL36 DUB is regulated by architectural rearrangements during infection.

## RESULTS

### Dynamic interactions of tegument proteins with capsids

**Fig 1:**
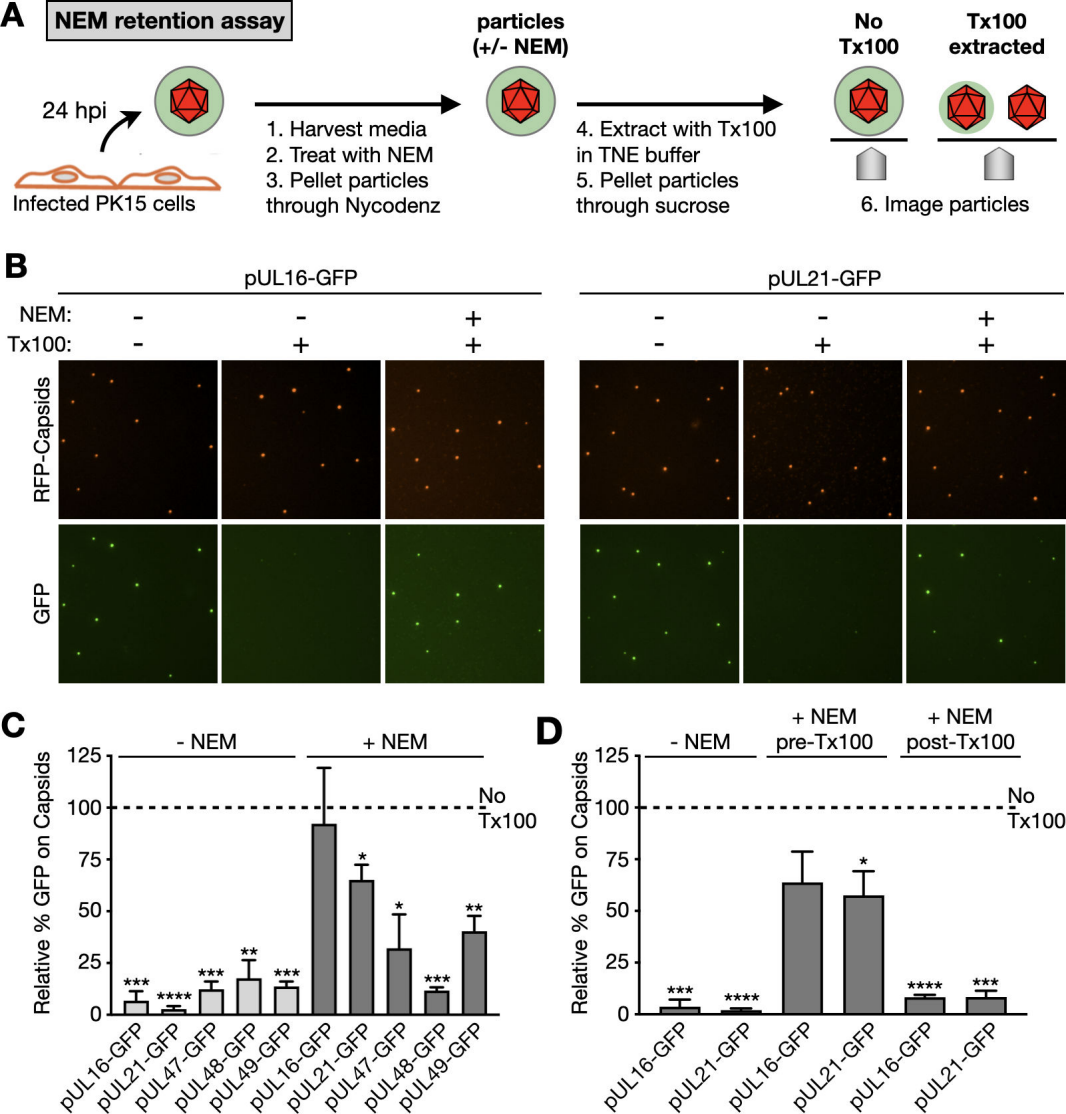
NEM-induced retention of selective tegument components on capsids using RFP–capsid/GFP–tegument dual-fluorescent PRV. (**A**) Experimental workflow of extracellular virus particle harvesting and processing in the NEM retention assay. (**B**) Representative images of extracellular virus particles processed in the NEM retention assay. (**C**) GFP emission from capsids processed by the NEM retention assay. Values were normalized to the cognate “No Tx100” control, which was set to 100% and represented by the dashed line (*n* = 3). (**D**) GFP emission from capsids processed by the NEM retention assay. NEM was either excluded (left), added prior to Tx100 extraction (middle), or added after Tx100 extraction (right). Values were normalized to the cognate “No Tx100” control, which was set to 100% and represented by the dashed line (*n* = 3). Error bars indicate standard deviation (*P < 0.05; **P < 0.01; ***P < 0.001; ****P < 0.0001 based on two-tailed unpaired *t* test with Welch correction).

N-ethylmaleimide (NEM) is a membrane-permeable alkene that covalently binds the thiol side chain of cysteine and prevents subsequent modifications (e.g., disulfide bond formation and acylation). Treatment of HSV-1 or PRV particles with NEM neutralizes their infectivity and inhibits the release of at least one tegument protein from the capsid upon disruption of the viral envelope. Specifically, the pUL16 tegument protein, which normally releases from capsids following extraction with nonionic detergents, remains capsid bound when viral particles are first treated with NEM (21, 22). NEM preservation of the pUL16-capsid interaction indicates that cysteine chemistry contributes to virion disassembly. To determine whether other tegument proteins have similar dynamic interactions with capsids, we adapted a fluorescence microscopy approach for assessing tegument–capsid association following NEM treatment and detergent extraction (referred to here as the NEM retention assay; Fig. 1A). PRV was modified to express pUL25/mCherry (red-fluorescent capsids) and green fluorescent protein (GFP) fused to a tegument protein: either pUL16, pUL21, pUL47, pUL48, or pUL49 (9, 36). These GFP fusions did not diminish viral titers relative to the parent red-fluorescent capsid strain (Table S2). Extracellular PRV particles were collected from the supernatant of infected PK15 cells at 18–22 hpi and were either treated with 10 mM NEM (+ NEM) or with an equivalent volume of ethanol (− NEM) for 30 min at 37°C. After removing cell debris by low-speed centrifugation, the particles were pelleted through a Nycodenz cushion by ultracentrifugation and then extracted with nonionic detergent (2% Triton X-100) in TNE buffer (20 mM Tris [pH 7.6], 500 mM NaCl, 1 mM EDTA). The latter solubilizes envelope and outer tegument components while retaining inner tegument proteins, including pUL36 and pUL37, on capsids. In this regard, this extraction procedure produces disassembled viral particles approximating the post-entry capsid complex that is liberated following cell entry (2, 3, 23, 37–39).

For each virus tested in the NEM retention assay, an unextracted sample (No Tx100) served as the baseline for tegument–GFP incorporation levels, an extracted sample not treated with NEM (− NEM) served as the measure of tegument–GFP released from capsids, and an extracted sample pretreated with NEM (+ NEM) tested for capsid retention. The viral particles from each sample were then pelleted through a 35% sucrose cushion to remove detergent and solubilized material. Pellets were resuspended in TNE buffer, and fluorescence microscopy was used to capture emissions from individual capsids (Fig. 1B). Diffraction-limited red and green fluorescence emissions from the capsids were quantitated using an image analysis algorithm (40). In the absence of detergent, particles from the five recombinant viruses displayed bright diffraction-limited red (capsid) and green (tegument) fluorescence. To quantitate the effect of detergent extraction on green emission levels, values for detergent-extracted samples were normalized to each corresponding unextracted sample (No Tx100), the latter of which was set to 100% across all virus preparations (Fig. 1C and D). Detergent extraction in the absence of NEM produced a significant loss of GFP from capsids with all viruses tested (Fig. 1C). In contrast, pretreatment with NEM retained pUL16–GFP on capsids, consistent with previous reports (21, 22). pUL21–GFP was also retained on capsids following NEM treatment and detergent extraction, although to a lesser extent than pUL16–GFP (Fig. 1B and C). NEM induced small increases in pUL47–GFP and pUL49–GFP capsid retention whereas pUL48–GFP retention was not increased (Fig. 1C). NEM-induced capsid retention of pUL16–GFP and pUL21–GFP only occurred when NEM was added prior to detergent extraction, suggesting that NEM prevents the release of an existing capsid interaction as opposed to causing re-binding (Fig. 1D).

### The N-terminus of pUL36 dynamically interacts with capsids

**Fig 2:**
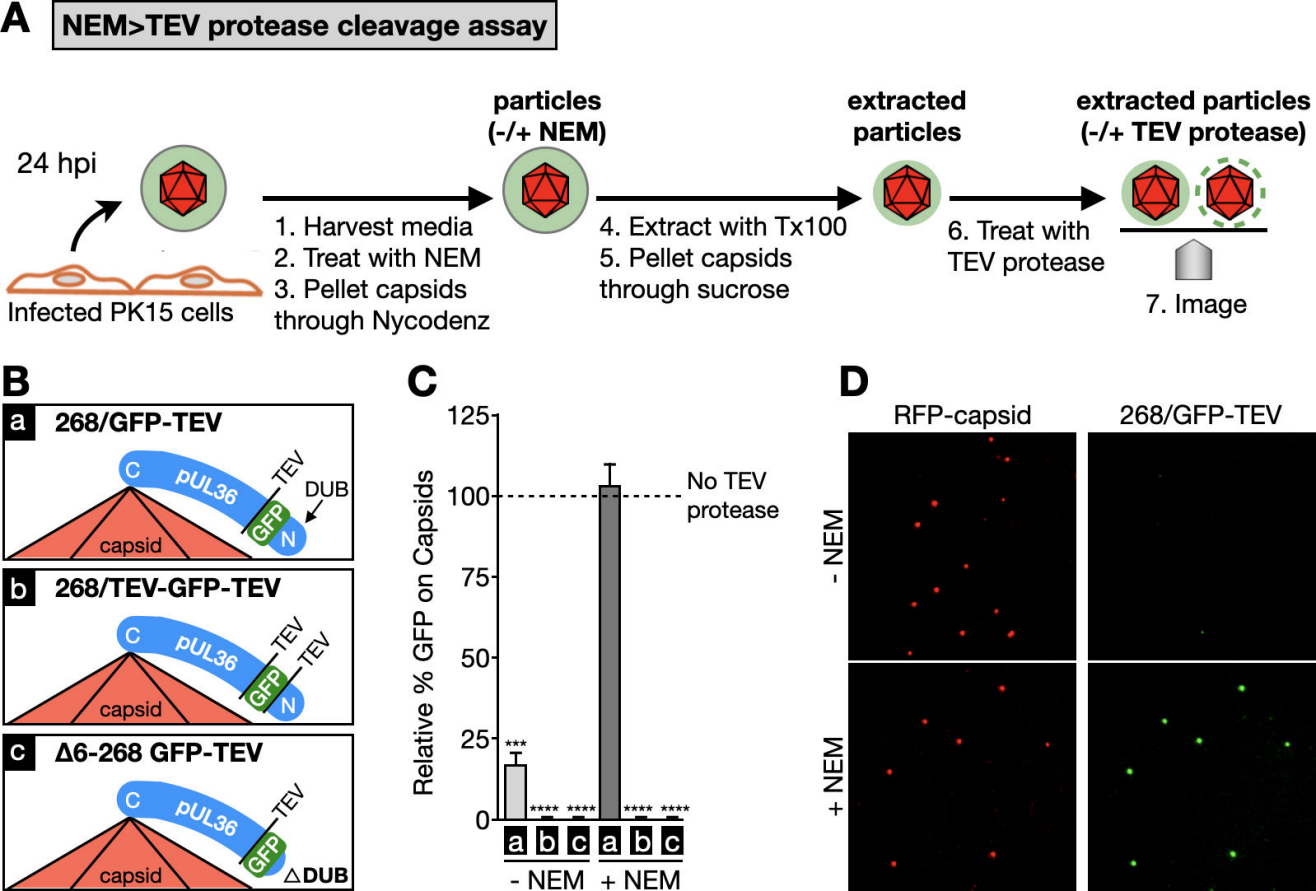
On-particle cleavage and release of pUL36 N-terminal fragments using RFP–capsid/GFP–pUL36 dual-fluorescent PRV. (**A**) Experimental workflow of extracellular virus particle harvesting and processing in the NEM >TEV protease cleavage assay. (**B**) Schematic of three PRV recombinants encoding one or two TEV protease cleavage sites at indicated GFP/pUL36 junctions. The ∆DUB recombinant (bottom) lacks amino acids 6–268 of pUL36. (**C**) GFP emissions from capsids processed by the NEM >TEV protease cleavage assay. Recombinant PRV used in the assay are as indicated in panel (B). Values for each sample are normalized to the cognate “No TEV protease” control, which is set to 100% and represented by the dashed line (*n* = 3). Error bars indicate standard deviation (***P < 0.001; ****P < 0.0001 based on two-tailed unpaired *t* test with Welch correction). (**D**) Representative images of the 268/GFP–TEV PRV particles processed in the NEM >TEV protease cleavage assay.

The large tegument protein, pUL36 (aka, VP1/2), is stably anchored to the capsid surface by its C-terminal CBD (26, 27, 41, 42). However, despite the remainder of pUL36 lacking stable capsid binding (37), there is indication that the pUL36–capsid interface may not be fully explained by the C-terminal CBD (35, 38, 43, 44). To investigate whether pUL36 possesses conditional CBDs, the NEM retention assay was adapted with the inclusion of Tobacco Etch Virus (TEV) protease cleavage site (ENLYFQG) to allow for site-specific cleavage and release of the N-terminus from the stable C-terminal CBD (Fig. 2A). To test this procedure, we first reexamined pUL16–GFP and pUL21–GFP capsid retention using derivatives of these viruses that encode a TEV site at the tegument–GFP junctions. Extracellular virus particles produced by the recombinant viruses were processed in the NEM-retention assay and then reacted with TEV protease (collectively referred to as the NEM >TEV protease cleavage assay). Green emissions from NEM-treated capsids were observed as before but were lost with TEV protease treatment, indicating that GFP was not retained by NEM while also validating the technical approach (Fig. S1).

Next, recombinant viruses were produced encoding pUL25/mCherry and GFP fused to pUL36 with a TEV site at the GFP–pUL36 fusion junction(s), with the intent that TEV protease treatment would liberate GFP from capsids by cleaving pUL36. This approach was initially pursued using recombinant viruses encoding GFP fused to the N-terminus of pUL36 but was abandoned upon discovering that NEM treatment prevented cleavage of the TEV site in this design (Fig. S2). This technical problem was overcome by encoding GFP as a double-in-frame fusion at codon 268 in pUL36 (Fig. 2B). This latter position was chosen for its low conservation within the alphaherpesviruses with the rationale that this site would be tolerant of insertions and accessible for TEV cleavage. Three viruses were produced based on this design encoding either one TEV site at the C-terminal GFP junction (268/GFP–TEV), TEV sites at both GFP junctions (268/TEV–GFP–TEV), or one TEV site at the C-terminal GFP junction that was also deleted for the pUL36 N-terminus (∆aa6–268 GFP–TEV).

Extracellular virus particles produced by each recombinant virus were processed in the NEM >TEV protease cleavage assay. In the absence of TEV protease, capsids from the three recombinant viruses emitted bright green fluorescence irrespective of NEM treatment at intensities that all were within twofold of each other, due to retention by the pUL36 C-terminal CBD. To quantitate the effect of cleavage on green emission levels, values for TEV-protease-treated samples were normalized to each corresponding untreated sample (No TEV protease), the latter of which was set to 100% across all virus preparations (Fig. 2C). In the absence of NEM, TEV protease liberated GFP from capsids for each virus. In contrast, following NEM treatment, TEV protease failed to release GFP from capsids of the 268/GFP–TEV virus (Fig. 2C and D). These results demonstrate that a conditional CBD is located in the pUL36 N-terminal region containing the DUB domain. Reinforcing this interpretation, TEV protease released GFP from capsids of the 268/TEV–GFP–TEV or ∆6–268 GFP–TEV recombinants. The 268/GFP–TEV recombinant served as the wild-type background strain for all subsequent recombinant PRV tested in this report, and in preparation for these studies, we note that insertion at aa268 did not impair PRV propagation (Fig. S3).

### The pUL16 and pUL21 tegument proteins are dispensable for pUL36 N-terminal capsid retention

To determine whether pUL16 or pUL21 contribute to pUL36 conditional capsid binding, the gene for each was deleted from the 268/GFP–TEV recombinant strain and tested in the NEM >TEV protease cleavage assay (Fig. 2A). NEM retained the pUL36 N-terminus on capsids for both viruses, although to a lesser degree than WT, and retention was insensitive to TEV protease. The results indicate that pUL16 and pUL21 were not required for N-terminal pUL36 capsid release or its retention but may play a minor role in the latter (Fig. S4).

### Cysteine residues in the DUB are required for capsid release

**Fig 3:**
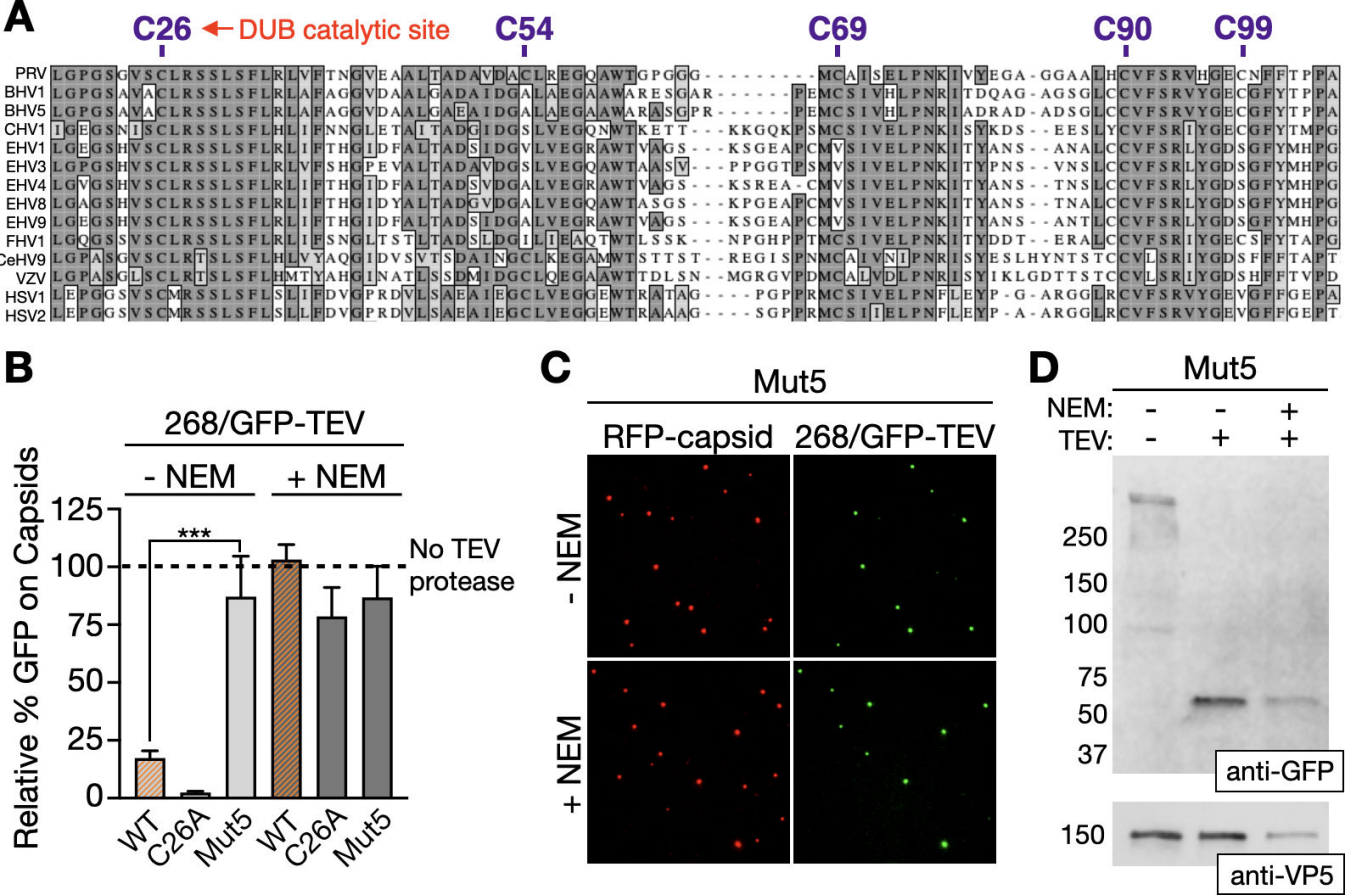
Mutation of cysteines in PRV pUL36 abrogates capsid release. (**A**) Alignment of the N-terminus of pUL36 across 14 members of the Alphaherpesvirinae subfamily. The five cysteines present in the first 268 amino acids of pUL36 are labeled at top with the amino acid position (only the first 106 amino acids are aligned because there are no cysteines in PRV pUL36 from aa107–268). Gray areas indicate regions of conservation. (**B**) GFP emissions from "268/GFP–TEV" capsids processed by the NEM >TEV protease cleavage assay. Each virus encodes RFP–capsids and GFP followed by a TEV protease cleavage site fused in-frame after aa268 of pUL36 (WT, no cysteines mutated; C26A, single mutation at catalytic site; Mut5, all five cysteines mutated). Values for each sample are normalized to the cognate “No TEV protease” control, which is set to 100% and represented by the dashed line (*n* = 3). WT data are duplicated from Fig. 2C (highlighted in orange). Error bars indicate standard deviation (***P < 0.001 based on ordinary one-way analysis of variance [ANOVA] followed by Dunnett’s multiple-comparison test). (**C**) Representative images of Mut5 virus particles processed in the NEM >TEV protease cleavage assay. (**D**) TEV protease cleavage of Mut5 viral particles examined by Western blot using an anti-GFP antibody and an anti-VP5 antibody as a loading control.

There are five cysteines in the first 268 amino acids of PRV pUL36, two of which are highly conserved (Fig. 3A). One of the latter, C26, is the cysteine protease catalytic residue of the DUB. To test if these residues contribute to capsid-binding dynamics, a PRV recombinant mutated for all five cysteines (Mut5) was produced. Mutation of the five cysteines is not predicted to cause larger changes to the DUB structure (Fig. S5). We also produced a PRV recombinant encoding a single C26A mutation (Table S1). Both viruses were tested in the NEM >TEV protease cleavage assay and compared to the prior parental 268/GFP–TEV results. The C26A derivative produced results equivalent to 268/GFP–TEV (WT), indicating that DUB activity did not confer the dynamic interaction between the pUL36 N-terminus and capsids (Fig. 3B). Similarly, TEV protease treatment did not release the Mut5 pUL36 N-terminus from capsids following NEM treatment, but interestingly, cleavage failed to release the N-terminus in the absence of NEM treatment (Fig. 3B and C). We confirmed that TEV protease cleaved Mut5 pUL36 by Western blot (Fig. 3D). The finding that the DUB cysteines promote capsid release is consistent with NEM inducing capsid retention and reinforces that cysteine chemistry is integral to the release mechanism. Furthermore, because the Mut5 N-terminal fragment lacks cysteines, these results rule out the possibility that a DUB–capsid disulfide bond was responsible for the attached state.

### The DUB is enzymatically inactive when tethered to the capsid

**Fig 4:**
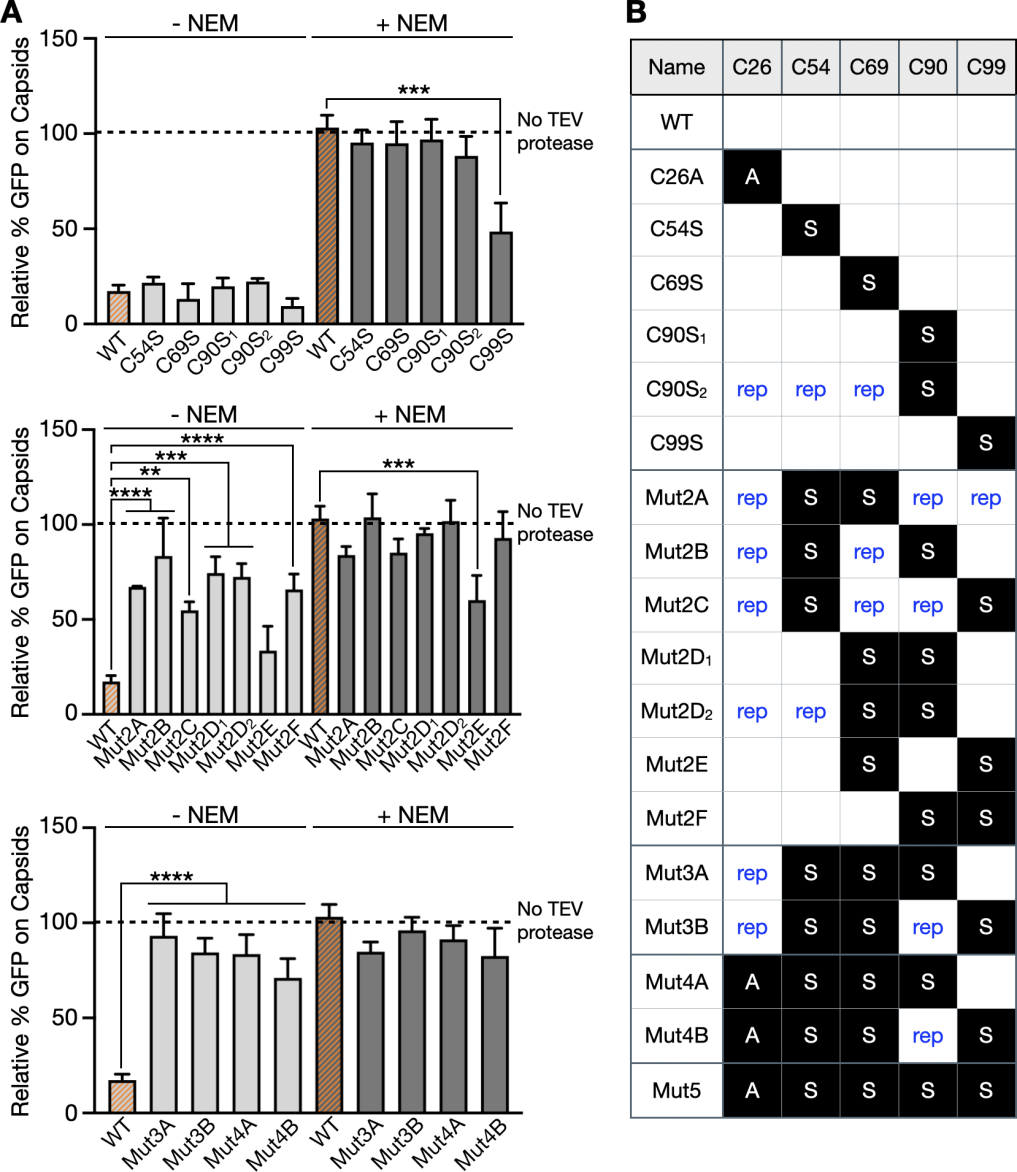
Extended mutational analysis of pUL36 DUB cysteines. (**A**) GFP emissions from "268/GFP–TEV" capsids processed by the NEM >TEV protease cleavage assay. Each virus encodes RFP–capsids and GFP followed by a TEV protease cleavage site fused in-frame after aa268 of pUL36. Values for each sample are normalized to the cognate “No TEV protease” control, which is set to 100% and represented by the dashed line (*n* = 3). WT data are duplicated from Fig. 2C (highlighted in orange). Error bars indicate standard deviation (**P < 0.01; ***P < 0.001; ****P < 0.0001 based on ordinary one-way ANOVA followed by Dunnett’s multiple-comparison test). (**B**) Summary of pUL36 cysteine mutants examined in this study. Mutations of the five cysteine residues are detailed (A, mutation to alanine; S, mutation to serine; rep, repaired to cysteine).

The finding that the pUL36 DUB dynamically binds to the capsid raised the question whether its enzymatic activity was regulated accordingly. Because PRV Mut5 possesses a C26A mutation that inactivates the enzyme, several combinations of cysteine mutations were introduced into the DUB region of PRV pUL36 to identify the critical residues for DUB release from the capsid. While no single cysteine mutation impaired capsid release, most combinations of two cysteine mutations were incapable of DUB release (the exception being Mut2E that encoded C69S/C99S), as were all combinations of three and four mutations tested. Duplicate mutants were made for C90S and Mut2D using different approaches and yielded consistent results (Fig. 4; Table S1).

**Fig 5:**
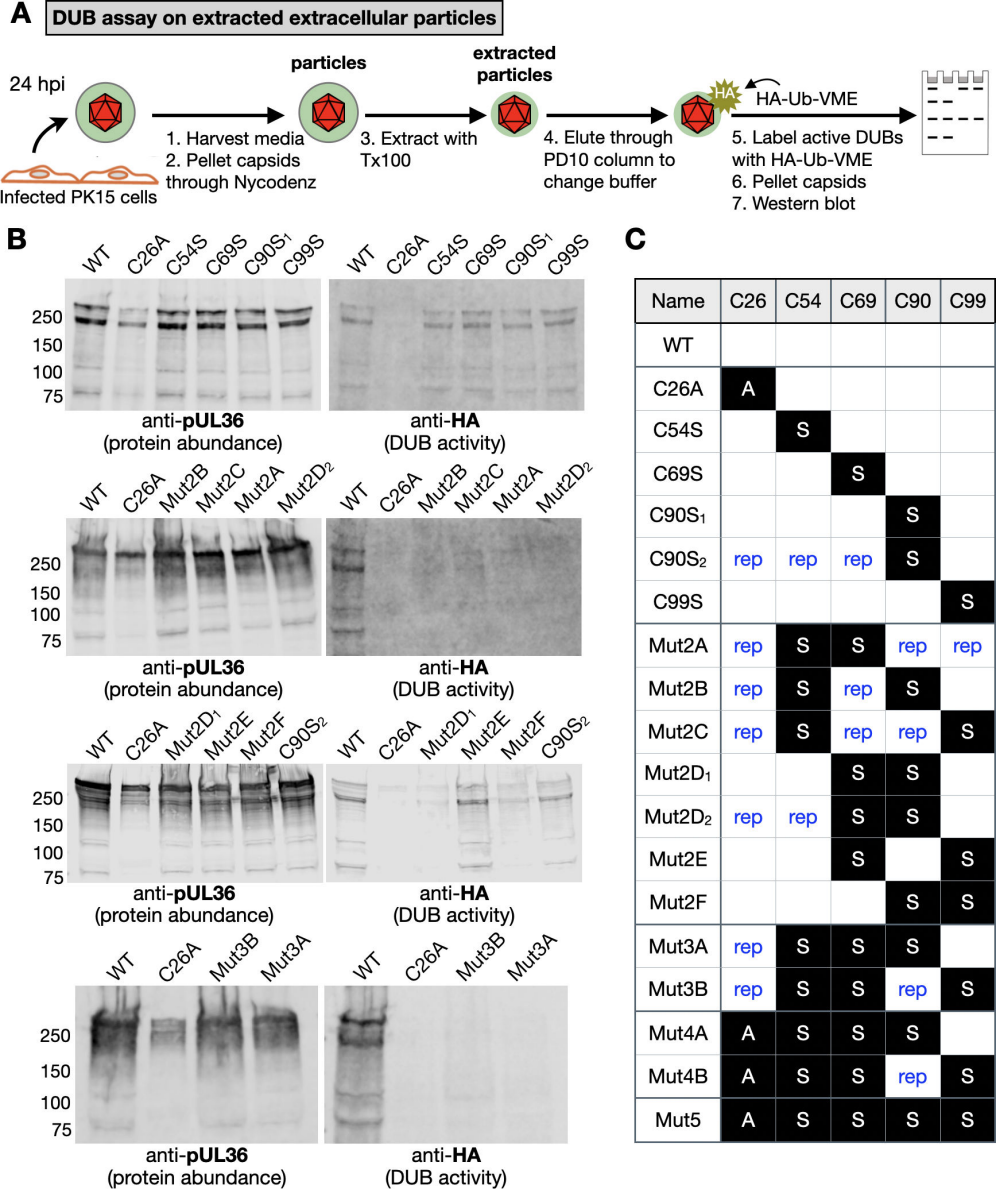
DUB catalytic activity in extracted PRV extracellular particles. (**A**) Experimental workflow of DUB assay using the HA-ubiquitin-vinyl methyl ester (HA-Ub-VME) probe. (**B**) Western blots of extracted extracellular viral particles processed in the DUB assay. An anti-pUL36 antibody was used to assess protein abundance, and an anti-HA antibody was used to detect labeling with the activity probe. (**C**) Summary of pUL36 cysteine mutants examined in this experiment. Mutations of the five cysteine residues are detailed (A, mutation to alanine; S, mutation to serine; rep, repaired to cysteine).

To determine whether the cysteine mutants had DUB activity, detergent-extracted extracellular viral particles were reacted with an HA-ubiquitin-vinyl methyl ester (HA-Ub-VME) that covalently binds to the C26 catalytic residue of the active DUB (45). The samples were run on SDS-PAGE and probed with anti-pUL36 and anti-HA antibodies (Fig. 5A). There was a correlation of DUB release with DUB enzymatic activity. PRV-encoding single-cysteine mutations, which all retain the capsid-release mechanism, were also enzymatically active: the one exception being mutation of the C26 catalytic site, which was competent for release but was, nevertheless, enzymatically inactive. Furthermore, all combination mutants that were defective for release also lacked enzymatic activity, and the one combination mutant that was release competent (Mut2E) was also enzymatically active (Fig. 5B; Table S1).

### DUB activity is suppressed in the capsid-bound state

**Fig 6:**
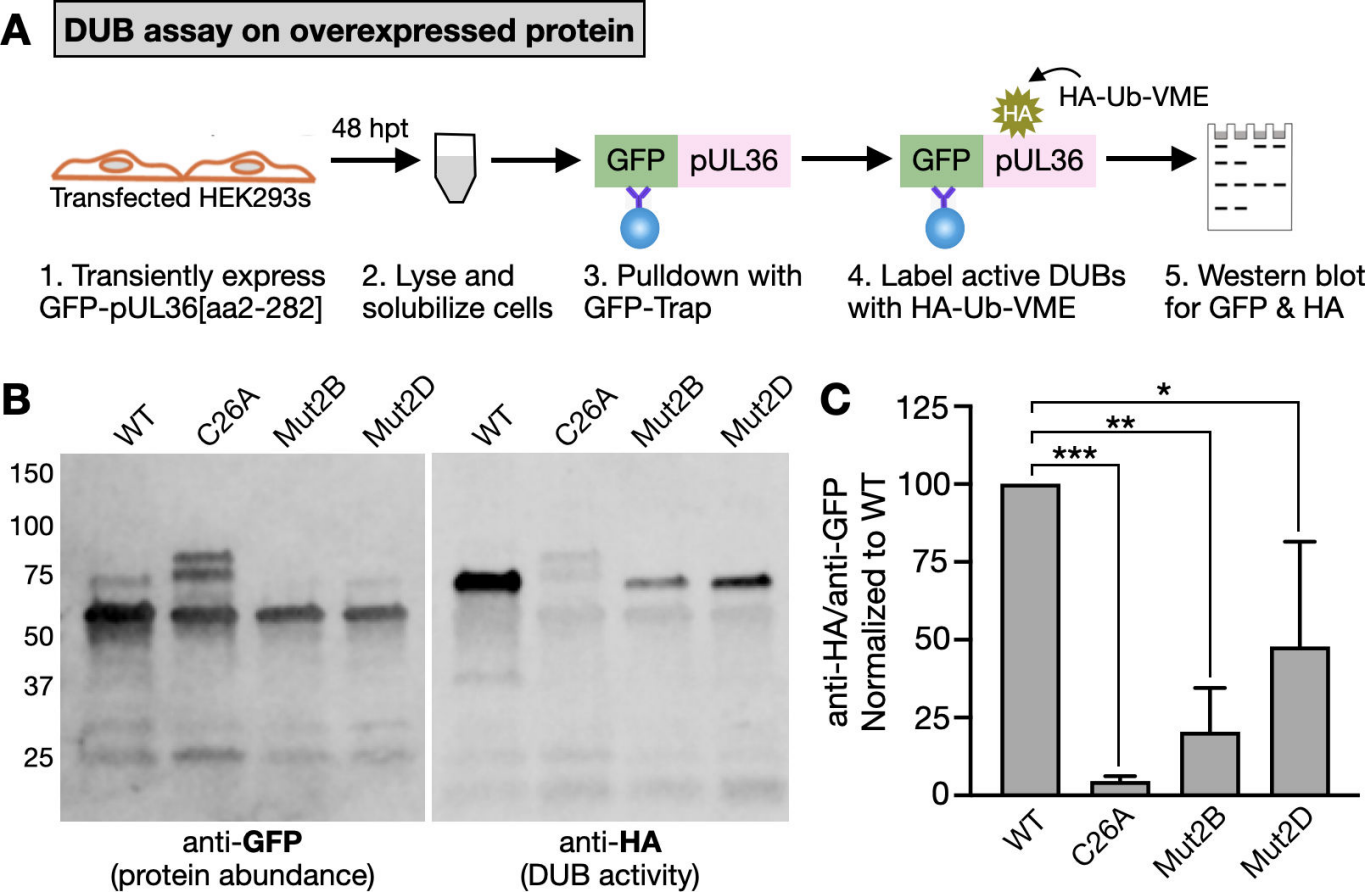
DUB catalytic activity in HEK293 cells transiently expressing GFP–pUL36[aa2–282]. (**A**) Experimental workflow of DUB activity assay on immunoprecipitated protein. (**B**) Transiently expressed WT and mutant GFP–pUL36 (amino acids 2–282) were immunoprecipitated from HEK293 cell lysates and labeled with the HA-Ub-VME DUB activity probe followed by Western blotting. An anti-pUL36 antibody was used to assess protein abundance, and an anti-HA antibody was used to detect labeling with the HA-Ub-VME activity probe. (**C**) Densitometry analysis of relative DUB activity (anti-HA signal relative to anti-GFP signal). Values are normalized to the WT average in each experiment (*n* = 3). Error bars indicate standard deviation (*P < 0.05; **P < 0.01; ***P < 0.001 based on ordinary one-way ANOVA followed by Dunnett’s multiple-comparison test).

Cysteines other than the catalytic site (C26 in PRV) were not previously implicated in DUB activity and are not part of the catalytic core of the enzyme (46). However, it remains possible that cysteine mutations impact DUB activity by altering the DUB conformation. Alternatively, the capsid may suppress the DUB in the bound state with the cysteines promoting DUB activity by triggering capsid release. To test these possibilities, Mut2B (C54S/C90S) and Mut2D (C69S/C90S), which lacked DUB activity in extracted extracellular viral particles, were transiently expressed in HEK293 cells as aa2–282 fragments fused to GFP. This allowed for testing enzymatic activity in the absence of capsids. A wild-type and C26A mutant served as positive and negative controls, respectively. Transfected cells were lysed, and the proteins were pulled down with GFP–Trap nanobodies prior to on-bead labeling with HA-Ub-VME (Fig. 6A). While only a fraction of WT pUL36 aa2–282 was labeled by HA-Ub-VME in this assay, this was clearly detectable because the probe increases the molecular weight of the DUB by ~8 kDa. We note that a greater fraction of the DUBs presumably could have been labeled using increased amounts of the costly HA-Ub-VME probe, but this was deemed unnecessary to measure relative activities. In contrast to the wild-type construct, the C26A control was not labeled by HA-Ub-VME; however, two unexpected bands (~75 and 80 kDa) appeared in the C26A sample that did not react with anti-HA antibody. We determined these species were ubiquitin conjugates that uniquely appeared when the DUB was inactive (Fig. S6; see Discussion). Regardless, both Mut2B and Mut2D were enzymatically active in the absence of capsids, although to a lesser degree than the wild-type control. Mut2D possessed the greatest activity (approximately 50% of WT), and this activity was in stark contrast to Mut2D when bound to capsids (compare Fig. 5 and 6). These results indicate that capsid-associated DUB activity is suppressed in the capsid-bound state.

### Neuroinvasion of pUL36 cysteine mutants

**Fig 7:**
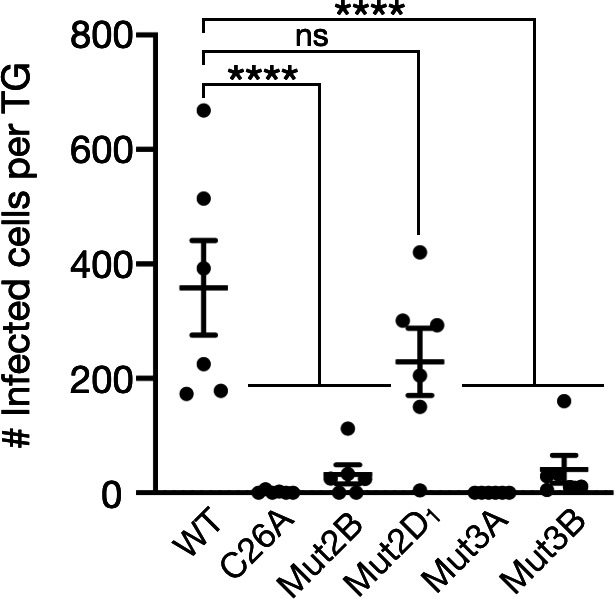
PRV invasion of the trigeminal ganglion. Mice were intranasally instilled with 1 × 10^5^ pfu of PRV per nostril (three mice per virus). Each virus encodes RFP–capsids and GFP followed by a TEV protease cleavage site fused in-frame after aa268 of pUL36. Both trigeminal ganglia (TG) were harvested at 48 hpi, and each was separately imaged for red fluorescence. Individual points represent the number of PRV-positive cells present in each harvested TG. Error bars indicate standard error of the mean (****P < 0.0001 based on ordinary one-way ANOVA followed by Dunnett’s multiple-comparison test).

PRV mutated in the catalytic C26 residue of the pUL36 DUB infects the nervous system with delayed kinetics following intranasal inoculation of mice (47). The DUB supports epithelial >neuron transmission but is dispensable for subsequent retrograde axonal transport (48). Consistent with this, PRV Mut5 was competent for retrograde axonal transport in cultures of primary sensory neurons and retained wild-type immediate early gene expression kinetics in epithelial cells (Fig. S7). To test if tethered release of the DUB from the capsid is required for neuroinvasion, infection of the trigeminal ganglia (TG) was examined following intranasal instillation of PRV into mice. As expected, PRV invasion of the TG was attenuated with the C26A mutant at 48 h post infection. Mut2D, which was enzymatically active when transiently expressed (Fig. 6), invaded the TG and was statistically equivalent to WT PRV (Fig. 7). Mut2B, which was less active than Mut2D when transiently expressed, was attenuated for TG invasion, as were Mut3A and Mut3B. Because these four mutants are locked onto capsids in an inactive state in viral particles, Mut2D reveals that the DUB enzymatic activity that specifically promotes neuroinvasion can come from pUL36 that is not associated with capsids and, conversely, that DUB activity associated with capsids is dispensable for neuroinvasion.

## DISCUSSION

The herpesvirus tegument consists of a complex assortment of viral and cellular proteins that are integral to the structure and the metastability of the virion. During viral assembly, inner tegument proteins are acquired on the capsid that bridge to outer tegument proteins and glycoproteins in the nascent envelope (1, 49–51). Upon initial infection, the outer tegument proteins dissociate, while the inner tegument proteins remain capsid bound and promote trafficking to the nucleus and injection of viral DNA through nuclear pores (2, 3, 6, 7, 31, 33–35, 44, 52–54). The dynamic interplay of assembly and disassembly is foundational to productive infection and allows for the continued propagation of virus from cell to cell.

The large inner tegument protein, pUL36, is essential both for virion assembly and for infectivity following virion fusion into cells (8, 33, 44, 47, 50, 51, 55). The protein is stably bound to the capsid by its C-terminal capsid-binding domain (CBD), which integrates with pUL17 and pUL25 to form the capsid vertex-specific component (CVSC) (27, 29, 30, 56). In the absence of pUL36, viral particles fail to assemble, and unenveloped capsids accumulate in the cytosol devoid of tegument (50, 51). While the pUL36 C-terminal CBD is critical for propagation of PRV and HSV-1 (35, 57, 58), it is not strictly essential for the production of fully assembled viral particles (e.g., H-particles) or their release from cells as might be expected. Instead, the function of the CBD is to retain pUL36 on capsids after entry into cells (35). Therefore, the interface of pUL36 with capsids must extend beyond the C-terminal CBD to fully account for its role in virion assembly.

Recent cryogenic electron microscopy (cryoEM) reconstructions of cytomegalovirus depict a complex interface between capsids and its pUL36 homolog (59), and work from other groups support the possibility that pUL36 binding to capsids could be multivalent (28, 35, 44, 60). Despite these reports, we were unable to detect regions of pUL36 that were stably bound to capsids following detergent extraction of PRV virions, apart from the C-terminal CBD (37). This raises the possibility that interactions between pUL36 and capsids that promote assembly are not preserved in virions upon envelope disruption.

Precedent for conditional capsid associations is established for the pUL16 tegument protein. Dynamic interaction between pUL16 and the capsid is based on cysteine reactivity, which we confirmed at the outset of this report (21, 22). Specifically, the membrane-permeant alkylating agent, NEM, prevents the release of pUL16, as well as pUL21, from capsids upon disruption of the viral envelope. The effect of NEM was specific in as much that it did not impair release of three other tegument proteins: pUL47 (aka VP13/14), pUL48 (aka VP16), and pUL49 (aka VP22). However, applying this approach to study pUL36 was not initially feasible because the protein is stably anchored to capsids via its C-terminal CBD; therefore, pUL36 should remain capsid bound irrespective of NEM treatment. To overcome this technical hurdle, recombinants of PRV were produced containing TEV cleavage sites in pUL36. Following virion assembly, these sites could be cleaved to liberate pUL36 fragments from the capsid-anchored C-terminal CBD. Combining NEM treatment with the TEV cleavage approach identified a conditional capsid-binding site in the pUL36 N-terminus.

**Fig 8:**
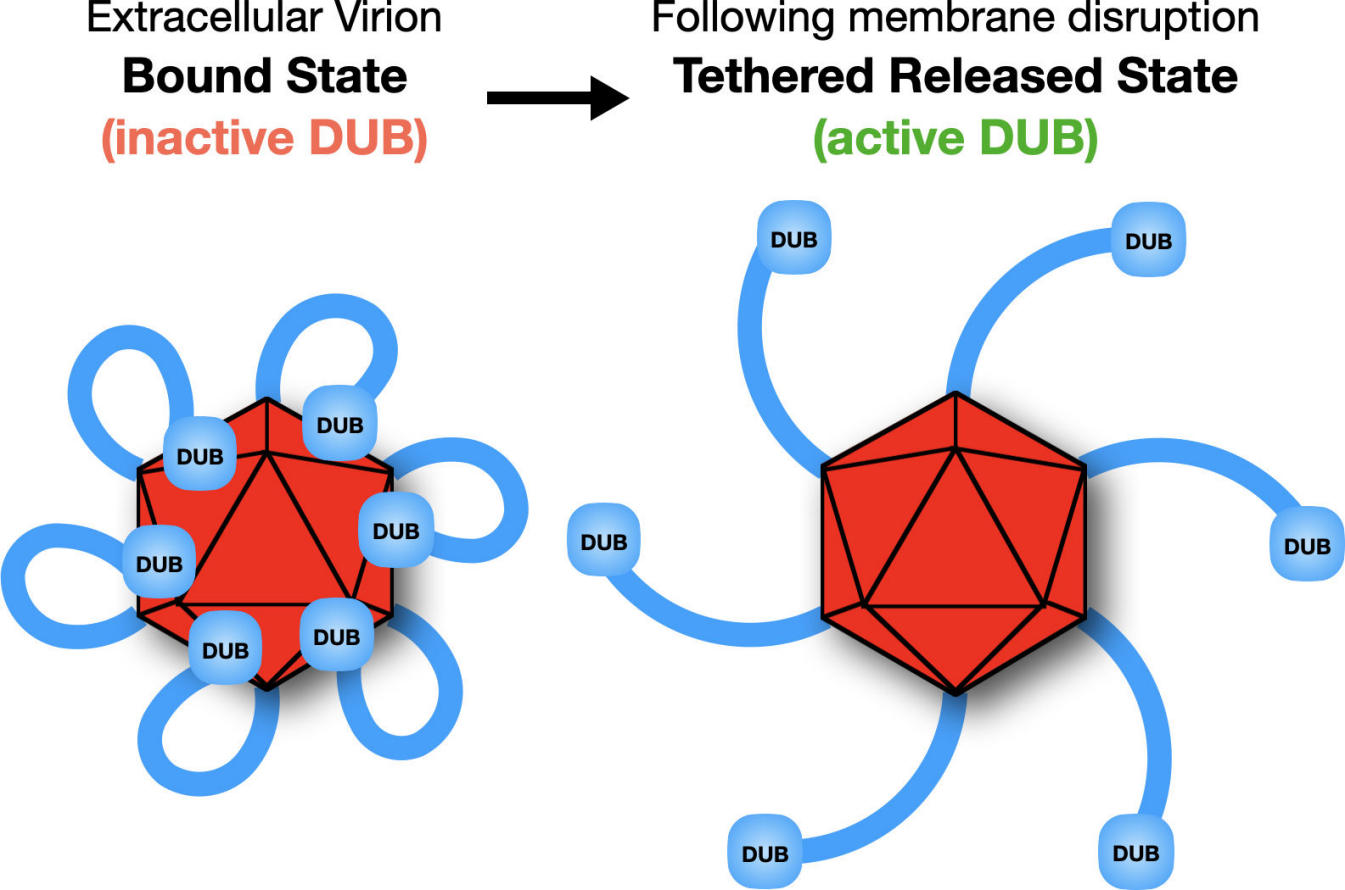
Model of pUL36 DUB regulation by conditional capsid binding. Whereas the N-terminal DUB of pUL36 binds capsids conditionally, the pUL36 C-terminus is stably bound to capsids as an integral element of the capsid-vertex specific component (CVSC). In the bound state, the DUB is catalytically inactive (left). Disruption of the virion envelope triggers DUB release from the capsid into a configuration in which the DUB is tethered to the capsid by the pUL36 C-terminus. The tethered configuration is expected to promote capsid-associated DUB enzymatic activity (right).

The N-terminus of pUL36 is the conserved herpesvirus deubiquitinase (DUB) (45, 61). The finding that NEM not only locked pUL16 and pUL21 onto capsids, but also the N-terminal DUB domain of pUL36, expands the implications for cysteine chemistry in virion dynamics. Consistent with reactive cysteines being required for DUB release from the capsid, mutation of the five cysteines within the PRV DUB converted it to a stable CBD in the absence of NEM. These results indicate that, like pUL16 and pUL21, the DUB undergoes triggered release from the capsid upon membrane disruption (Fig. 8). Further work will be needed to elucidate the specific contributions of the DUB cysteines in capsid association; however, a few points can be made. First, while there are several post-translational modifications that can occur on free cysteines, the ability of NEM treatment to prevent DUB release from extracellular virions indicates that at least some of the DUB cysteines are unmodified in virions. Therefore, oxidation–reduction (ox-redox) of cysteines is likely at play. We can safely rule out that a disulfide bond links the DUB to the capsid because NEM does not impact preexisting disulfide bonds, and furthermore, mutation of the DUB cysteines did not prevent the DUB–capsid interaction but instead produced the opposite effect of locking it in place. These observations may be accounted for by cysteine isomerization, in which DUB cysteines normally attack a preexisting disulfide bond elsewhere in the virion. AlphaFold modeling of the pUL36 N-terminus showed only C26 having the sulfhydryl side chain facing outward, suggesting the other four cysteines may engage in intramolecular interactions. Although intramolecular disulfide bonds were not predicted in the AlphaFold model, different DUB conformations could occur during infection that allow for either intra- or inter-molecular disulfide isomerization reactions (Fig. S5). Although presumably unrelated to this ox-redox reactivity, we also fortuitously discovered that the GFP–DUB fusion protein was ubiquitinated when catalytically inactive, indicating that it recruits an E3 ligase. This finding is noteworthy because DUBs and E3 ligases often work as a complex (62), and pUL36 ubiquitination was previously reported to support retrograde axonal invasion of the nervous system (52).

Given the findings that the pUL36 DUB undergoes capsid binding and releases by a cysteine-based mechanism, we were interested to learn whether DUB enzymatic activity, which is based on a catalytic cysteine protease core, might be regulated by capsid binding. This hypothesis could not be tested with NEM, which inactivates the C26 catalytic cysteine of the DUB, nor could Mut5 be used for similar reasons. Instead, a series of PRV-encoding cysteine mutations in the pUL36 DUB were examined, revealing that viruses with a functional DUB release mechanism retained DUB enzymatic activity, whereas viruses that failed to release the DUB from capsids coincidentally lacked DUB activity. Interestingly, two such mutants, Mut2B (C54S + C90S) and Mut2D (C69S + C90S), lacked DUB activity in virions but had DUB activity when transiently expressed independently from capsids. Because DUB catalytic activity promotes epithelial-to-neuronal transmission of PRV in mice, we were curious if Mut2B and Mut2D retained this property (48). Mut2D, unlike a C26A catalytic mutant, remained competent to invade the trigeminal ganglion (TG) following intranasal instillation. From this we infer that DUB release from capsids, which likely occurs when virus initially enters cells, is not essential for neuroinvasion. In addition, this finding suggests that a capsid-free population of DUB can promote neuroinvasion.

Our finding that the DUB is inactive in the capsid bound state and is activated by its release from capsids demonstrates that the DUB is regulated by architectural rearrangements during infection. While these findings suggest the DUB can be turned on or off during different stages of infection, it remains possible that non-capsid-bound pUL36 could retain DUB activity throughout infection and be regulated by different mechanisms. For instance, capsid-associated DUB from Mut2D was inactive in extracellular virions (when bound to the capsid) but could be active when free of the capsid. In fact, the finding that Mut2D promoted neuroinvasion, which is dependent on DUB activity, indicates that DUB activity from Mut2D may occur during late infection before becoming bound to the capsid or be provided by a population of pUL36 that never associates with capsids. In support of the latter, studies have reported that multiple forms of pUL36 are expressed during late infection, including C-terminal truncations that presumably lack stable capsid binding (35, 38, 43, 44). Our finding that the DUB is active in detergent-extracted virions, which mimics the post-entry state, is consistent with the DUBs’ role in overcoming interferon-induced cellular resistance to initiate lytic replication upon entry (63). Furthermore, there are likely additional levels of DUB regulation that restrain autocatalytic activity given that pUL36 ubiquitination at K442 supports retrograde axonal transport and neuroinvasion (52), which may relate to the fortuitous finding that the DUB recruits a cellular E3 ligase activity.

In conclusion, this study documents that the pUL36 large tegument protein, which is an essential structural and infectivity component of virions, alters its association with capsids based on cysteine reactivity that underlies virion metastability while also modulating its deubiquitinase activity.

## MATERIALS AND METHODS

### Sequence alignment

Predicted amino acid sequences of pUL36 alphaherpesvirus homologs were aligned using SnapGene software. GenBank accession numbers used were JF797219 (pseudorabies virus [PRV]), AJ004801 (bovine herpesvirus 1 [BHV1]), NC_005261 (bovine herpesvirus 5 [BHV5]), ARE29826 (canid alphaherpesvirus 1 [CHV1]), YP_053069 (equid herpesvirus 1 [EHV1]), YP_009054927 (equid herpesvirus 3 [EHV3]), NP_045241 (equid herpesvirus 4 [EHV4]), YP_010795064 (equid herpesvirus 8 [EHV8]), YP_002333505 (equid herpesvirus 9 [EHV9]), WOV89978 (felid herpesvirus 1 [FHV1]), NP_077437 (cercopithecine alphaherpesvirus 9 [CeHV9]), NC_001348 (varicella-zoster virus [VZV]), GU734771 (herpes simplex virus 1 [HSV-1]), and NC_001798 (herpes simplex virus 2 [HSV-2]).

### BAC mutagenesis

Recombinant PRV strains used in this study were constructed by two-step, red-mediated recombination with the pBecker3 infectious clone (64, 65). A summary of the recombinant strains of PRV used in this study are provided in Table S2, and the primers used for their production are listed in Table S3. To create PRV-GS7235 and PRV-GS7521, tobacco etch virus (TEV) protease recognition sites (ENLYFQG) were inserted into the pBecker3 derivative, pGS1968, after amino acid 268 of pUL36. To create the fluorescent PRV strains used in this study, TEV protease recognition sites and the GFP coding sequence were inserted into the pBecker3 derivative, pGS4284, which encodes for a pUL25/mCherry fusion (36). Deletion of pUL16, deletion of pUL21, and cysteine mutagenesis of pUL36 were performed on the pBecker3 derivative of pGS7613, which encodes pUL25/mCherry and pUL36 fused to GFP, a TEV protease recognition site, and a Gly–Gly–Gly linker that were inserted in tandem after amino acid 268 of pUL36 (referred to here as 268/GFP–TEV).

### Cells and viruses

PK15 epithelial cells were maintained in Dulbecco’s modified Eagle medium (DMEM, Gibco) supplemented with 10% (vol/vol) bovine growth serum (BGS, HyClone). Infectious clones were transfected into PK15 cells as previously described to produce stocks of recombinant PRV (2). The resulting viral stocks were passaged once through PK15 cells to make working stocks. During infection, the BGS concentration was reduced to 2% (vol/vol). Titers of virus stocks were determined by plaque assay on PK15 cells as previously described (66). Individual plaque diameters on PK15 cells were measured from the average of two orthogonal cross sections based on pUL25/mCherry fluorescence emissions. Approximately 30 plaques were measured to determine the average plaque size for each virus, which was normalized as a percentage of the parental virus plaque diameter.

### Preparation of extracted viral particles for NEM retention assay

PK15 cells in 10-cm dishes were infected with PRV strains at an MOI of 10 pfu/cell and incubated for 18–22 h in DMEM supplemented with 2% (vol/vol) BGS. Supernatants were placed in 15-mL conicals and were either treated with 10 mM N-ethylmaleimide (NEM, Millipore Sigma) prepared in ethanol (1 M stock of NEM was made fresh for each experiment) or an equivalent volume of ethanol, and were incubated at 37°C for 30 min. Supernatants were then cleared of cell debris with a 10-min 3,000 × *g* centrifugation in 15-mL conical tubes at 4°C. Next, 9 mL of cleared supernatant was placed in SW41 tubes (Seton #7030) and underlaid with a 1-mL cushion of 10% (wt/vol) Nycodenz (Accurate Chemical) in TNE buffer (20 mM Tris [pH 7.6], 500 mM NaCl, 1 mM EDTA). Viral particles were pelleted through the Nycodenz by centrifuging for 1 h at 15,000 rpm at 4°C in an SW41 rotor. Media and the Nycodenz cushion were removed by aspiration, and the pellets were dispersed by gentle pipetting in 0.5 mL of TNE with ±2% Triton X-100 (Tx100) for a minimum of 20 min on ice. Both Tx100-extracted and intact particles were diluted in 4 mL of TNE buffer and overlayed onto a 5-mL 35% sucrose cushion in TNE buffer in SW41 tubes. The samples were centrifuged for 1 h at 25,000 rpm in an SW41 rotor at 4°C. The pellet was resuspended in 100 µl of TNE and dispersed with gentle pipetting. For light microscopy, the intact or extracted particles were diluted with TNE and imaged on a plasma-treated no. 1.5, 22- by 22-mm coverslip in a microscope slide chamber sealed with 1:1:1 mixture of petrolatum, lanolin, and beeswax (VALAB).

### Preparation of viral particles for NEM > TEV protease cleavage assay

PK15 cells in 15-cm dishes were infected with PRV strains at an MOI of 3 pfu/cell and incubated for 18–22 h in DMEM supplemented with 2% (vol/vol) BGS. Approximately 32 mL of supernatants was placed in 50-mL conicals and were either treated with 10 mM NEM (from freshly made 1 M NEM stock prepared in ethanol) or an equivalent volume of ethanol and were incubated at 37°C for 30 min. Supernatants were then cleared of cell debris with a 20-min 5,000 × *g* fixed-angled centrifugation in 50-mL conical tubes at 4°C. Next, 32 mL of cleared supernatant was placed in SW32 tubes (Seton #7052) and were underlaid with a 3-mL cushion of 10% (wt/vol) Nycodenz in TNE buffer (20 mM Tris [pH 7.6], 500 mM NaCl, 1 mM EDTA). Viral particles were pelleted through the Nycodenz by centrifuging for 1 h at 15,000 rpm at 4°C in an SW32 rotor. Media and the Nycodenz cushion were aspirated from pelleted virus, and the pellets were dispersed by gentle pipetting in 0.5 mL of TNE with 2% Tx100 for a minimum of 20 min on ice. Extracted particles were diluted in 4 mL of TNE buffer and overlayed onto a 5-mL 35% sucrose cushion in TNE buffer in SW41 tubes. The samples were centrifuged for 1 h at 25,000 rpm in an SW41 rotor at 4°C. The pellets were resuspended in 100 µL of TEV resuspension buffer (25 mM Tris [pH 8.0], 150 mM NaCl) and dispersed with gentle pipetting. Dithiothreitol (DTT; 1 mM) was added to each viral particle resuspension before splitting each evenly into two microcentrifuge tubes with one tube receiving 5 µL of TEV protease in storage buffer (Millipore Sigma) or an equal volume of TEV protease storage buffer lacking enzyme (25 mM Tris-HCL [pH 8.0], 50 mM NaCl, 1 mM TCEP, 50% (vol/vol) glycerol) followed by incubation at 30°C in the dark for 18 h. Following the incubation, particles were diluted and dispersed in TEV resuspension buffer for imaging by fluorescence microscopy on a plasma-treated no. 1.5, 22- by 22-mm coverslip in a VALAB-sealed microscope slide chamber. In some cases, the processed particles were pelleted at 12,000 rpm for 30 min at 4°C, and the aspirated pellets were combined with 20 µL of 2× final sample buffer (62.5 mM Tris [pH 6.8], 10% glycerol, 2% SDS, 0.01% bromophenol blue) for Western blot analysis.

### Quantitative fluorescence microscopy

Viral particles were imaged on an inverted wide-field Nikon Eclipse TE2000-U microscope fitted with a 60 × 1.4 numerical aperture objective, an Evolve electron-multiplying charge-coupled device (EM-CCD) camera (Photometrics), and a Lumen PRO Fluorescence Illumination System (Prior Scientific) set at 100% light output. Enhanced GFP was imaged using a 470/40-nm excitation filter and a 525/50-nm emission filter; mCherry fluorescence was imaged using a 572/35-nm excitation filter and a 632/60-nm emission filter (Chroma Technology Corporation). Red and green fluorescence was captured using 1-s exposures, with a 1-MHz digitizer and zero electron multiplying gain. Fluorescent intensities from individual particles were quantified with an automated custom algorithm as previously described (40). Briefly, the algorithm identified diffraction-limited red-fluorescent punctae consistent with individual capsids. Red and green intensity measurements from the viral particles were compiled from three independent experiments, with >100 particles measured per experiment. Red-fluorescent capsids from the NEM > TEV protease cleavage assay with intensities outside 2 standard deviations of the mean were postfiltered from the data sets to remove noise and virion clusters. Red-fluorescent capsid intensities from intact viral particle preparations and extracted viral particle preparations from the NEM retention assay were plotted as histograms, where the Freedman–Diaconis rule was applied to independently determine bin sizes (67). Nonlinear Gaussian regression was performed in GraphPad Prism, and red-fluorescent capsids with intensities outside 3 standard deviations of the mean were removed to avoid noise and virion clusters. In all cases, the remaining green-fluorescence intensities were normalized to their corresponding controls and plotted on bar graphs. For the NEM retention assay, green-fluorescence intensities for the Tx100-treated samples were normalized to their corresponding no Tx100 control intensities, and statistical significance was determined by a *t* test with Welch correction comparing each Tx100-treated sample to the no Tx100 control. For the NEM > TEV protease cleavage assays done with WT pUL36 (no cysteine mutations or tegument deletions), green-fluorescence intensities for the TEV protease-treated samples were normalized to their corresponding no TEV protease control intensities and statistical significance was determined by a *t* test with Welch correction comparing each TEV protease treated sample to the no TEV protease control. For NEM >TEV protease cleavage assays where mutant strains are compared to WT, green fluorescence intensities for the TEV protease-treated samples were normalized to their corresponding no TEV protease control intensities, and statistical significance was determined by one-way ANOVA with multiple comparison test.

### Virus gene expression

PK15 cells were grown to confluence in six-well plates and infected with PRV at a MOI of 1 pfu/cell. Total RNA was isolated at 4 hpi with PureLink RNA Mini Kit (Invitrogen) according to the manufacturer’s instructions. Contaminating DNA was removed with on-column PureLink DNase treatment (Invitrogen) during the RNA isolation. RNA concentration was determined by absorbance at 260 nm with a NanoDrop 8000 (Thermo Scientific). Gene-specific complementary DNA (cDNA) was synthesized using the Applied Biosystems High-Capacity cDNA Reverse Transcription Kit (ThermoFisher Scientific). The final RNA concentration was diluted to 50 ng/µL and combined with 10 µL of cDNA master mix to form cDNA with run settings of 25°C for 10 min, 37°C for 120 min, and 85°C for 5 min. cDNA was diluted 1:5 in nuclease-free water to a final concentration of 5 ng/µL. SYBR Green-based quantitative real-time PCR was performed on cDNA in a LightCycler 480 II (Roche). All reactions were carried out in 20-µL volumes: 5 µL of cDNA, 10 µL of LightCycler 480 SYBR Green I Master (Roche), 2 µL of 10× forward primer and 2 µL of 10× reverse primer (0.5 µM each), and 3 µL of nuclease-free water. Each sample was run in triplicates, and a control sample lacking reverse transcriptase enzyme was included in parallel. The run settings were 95°C for 5 min, 50 cycles of 95°C for 10 s, 60°C for 20 s, and 72°C for 20 s. The forward and reverse primer sequences were as follows: PRV IE180 forward, CATCGTGCTGGACACCATCGAG; PRV IE180 reverse, ACGTAGACGTGGTAGTCCCCCA; pig 28S forward, GGGCCGAAACGATCTCAACC; pig 28S reverse, GCCGGGCTTCTTACCCATT. Specificity of primer binding was confirmed by melting point analysis. IE180 mRNA levels were normalized to S28 rRNA. Fold change was calculated using the threshold cycle (2ΔΔCT) method, and the fold change between WT and Mut5 PRV infections was averaged across four independent experiments. Statistical significance was determined by a *t* test with Welch correction.

### Primary neuronal culture

Dorsal root ganglia (DRG) from embryonic chicks (E8-E10) were cultured on poly-DL-ornithine and laminin-treated coverslips in 2 mL of F12 medium (Invitrogen) containing nutrient mix: 0.08 g/mL of bovine serum albumin fraction V powder (VWR), 0.4 mg/mL of crystalline bovine pancreas insulin (Sigma-Aldrich), 0.4 µg/mL of sodium selenite (VWR), 4 µg/mL of transferrin (Intercell Technology), and 5 ng/mL of nerve growth factor (NGF; Sigma-Aldrich). A single DRG explant was cultured on each coverslip for 2 to 3 days and infected with 2 × 10^7^ PFU of PRV. The infections were incubated for 5 min at 37°C before mounting to slides for time-lapse fluorescent microscopy.

### Time-lapse fluorescence microscopy and image analysis

DRG on coverslips were mounted to glass slides and sealed with VALAB to produce closed chambers containing approximately 70 µL of media, and multiple time-lapse image series of mCherry emissions were captured during 0.5 to 1.0 hpi at 10 frames/s for 200 frames each. Images were captured using an inverted wide-field Nikon Eclipse TE2000-U microscope fitted with a 60 × 1.4 numerical aperture objective and an Evolve EM-CCD camera (Photometrics). The microscope was housed in a 37°C environmental box (*In Vivo* Scientific). Illumination was provided by a Lumen PRO Fluorescence Illumination System (Prior Scientific) set at 100% light output. Particle trajectories were traced in the 200-frame time-lapse image series using a multiline tool with a width of 20 pixels and average background subtraction, and a kymograph was produced using the MetaMorph software package (Molecular Devices). The multiline tool was used to trace kymograph paths, which were used to calculate individual capsid transport velocities, run lengths, and net displacement with filtering for distances of <0.5 µm to filter out random diffusion of virions. Runs were defined as uninterrupted diagonal lines (distance/time) and were measured for the distance traveled in the retrograde direction (length) and the average retrograde velocity (slope). Greater than 100 particles were analyzed per virus across three replicate experiments, and an average value was calculated for each virus. Values reported represent the mean ± standard deviation of the average values obtained. Significance was determined using Student’s two-tailed unpaired *t* test.

### Assaying DUB activity in viral particles

PK15 cells in 15-cm dishes were infected with PRV strains at an MOI of 3 pfu/cell and incubated for 18–22 h in DMEM supplemented with 2% (vol/vol) BGS. Approximately 32 mL of supernatants was placed in 50-mL conicals and were then cleared of cell debris with a 20-min 5,000 × *g* fixed-angled centrifugation at 4°C. Next, 32 mL of cleared supernatant was placed in SW32 tubes and were underlaid with a 3-mL cushion of 10% (wt/vol) Nycodenz in TNE buffer (20 mM Tris [pH 7.6], 500 mM NaCl, 1 mM EDTA). Viral particles were pelleted through the Nycodenz by centrifuging for 1 h at 15,000 rpm at 4 °C in a SW32 rotor. Media and the Nycodenz cushion were removed by aspiration, and the pellets were dispersed by gentle pipetting in 0.5 mL of TNE +2% Tx100 for a minimum of 20 min on ice. Each sample was passed through an equilibrated PD MiniTrap^TM^ G-25 column (Cytiva) and eluted in 1 mL of HA-Ub-VME labeling buffer (50 mM Tris [pH 7.4], 5 mM MgCl, 0.5 mM EDTA, 2 mM DTT, 2 mM ATP, 250 mM sucrose). HA-Ub-VME probe (Enzo Life Sciences; 3 µg from a 1 µg/µL stock in 50 mM Tris [pH 7.6] and 150 mM NaCl) was added to each 1 mL of viral particle resuspension and rotated at room temperature for 1 h. Viral particles were pelleted by centrifugation at 12,000 rpm for 10 min at 4°C. Aspirated pellets were resuspended in 25 µL of 2× final sample buffer (62.5 mM Tris [pH 6.8], 10% glycerol, 2% SDS, 0.01% bromophenol blue) for Western blot analysis.

### Plasmid construction

Four plasmid constructs encoding amino acids 2–282 of PRV pUL36 with in-frame N-terminal strep-tags were synthesized by Twist Bioscience. The constructs encoded for either the wild-type allele (pGS7834), a C26A mutant allele (pGS7835), a C54S/C90S mutant allele (pGS7836), or a C69S/C90S mutant allele (pGS7837). All were flanked by HindIII and EcoRI restriction sites. Each allele was subcloned into pEGFP-C3 to produce GFP-strep-pUL36[aa2–282] fusions for mammalian expression, resulting in pGS7900, pGS7901, pGS7902, and pGS7903, respectively. Each construct was sequence confirmed.

To generate the constructs used in Fig. S6, the UL36 ORF was subcloned from the pBecker3 infectious clone using RED-GAM protocols. To accomplish this, a start methionine codon, with an optimized Kozak sequence followed by a strep-tag encoding sequence and Gly-Ser linker, was inserted in front of codon 2 of pUL36 along with a pGS1292 R6K plasmid. The plasmid was first amplified with the primers 5′ CCAAATAAAAAGATTTTTCCCCCACGCGCGTGTGTTATTTCAGCCGATTTTTATC**GA****ATTC**GTCATCCATATCACCACG and 5′ TACTGATTACGATAGCCGACGACCACCGCGTCGGCCGT*GCTGCCCTTTTCGAACTGCGGGTGGCTCCACGCCATGGT***AAGCTT**CCACATGTGGAATTCCC (linker, strep-tag, and Kozak sequences are in italics, EcoRI and HindIII sites are in bold, and pGS1292 homologies are underlined). The resulting PCR product was inserted upstream of the UL36 start methionine codon based on homologies in the 5′ end of each primer and selecting for an amp-resistant recombinant. This resulted in pGS3260. Next, a stop codon followed by an EcoRI site was inserted downstream of codon 282 in UL36, by PCR amplifying a kan-resistance cassette off of template plasmid pEP–GFP-in using primers 5′ CCGCTCCCGCCGCCGCCGCGGGTCCAGAAGCGGCCGAGC*TAA***GAATTC**GTGAGCAAGGGCGAGGAG and 5′ ACCGCAGAGGCAAACAAGTTGGGTAATAAACAATTTATTACTTGTACAGCTCGTCCATGCCG (stop codon is in italics, EcoRI site is in bold, and pEP–GFP-in homologies are underlined), and selecting for a kan-resistant recombinant. This resulted in pGS3261. The UL36 N-terminal coding fragment was liberated by EcoRI digestion and self-ligation between the EcoRI sites engineered both upstream of the pGS1292 insertion and downstream of aa282 codon in UL36 and was transformed into *Escherichia coli* strain S17 lambda-pir, resulting in pGS3285. The strep-UL36 [aa2–282] encoding insert was moved into pEGFP-C3 from pGS3285 as an EcoRI–HindIII fragment resulting in plasmid pGS3295, which encodes GFP–strep–UL36 [aa2–282] behind a CMVIE promoter. This process was repeated with a derivative of pBecker3 encoding pUL36 with a C26A mutation (pGS1652). For C26A, the amp-resistant intermediate was pGS3454, the Kan-resistant intermediate was pGS3450, the intermediate with the UL36 N-terminal coding fragment liberated by EcoRI digestion, and self-ligation was pGS3477, and the final expression construct was pGS3482.

### Immunoprecipitation of GFP-strep-UL36 [aa2–282]

HEK293 cells seeded in 10-cm plates were transfected with pEGFP-C3 or derivatives encoding GFP–strep–pUL36 [aa2–282], either WT (pGS3295) or C26A (pGS3482), using polyethylenimine (Polysciences) as follows: 1.5 mL of DMEM, 225 µL of polyethylenimine solution (1 mg/mL), and 25 µg of plasmid DNA were added, mixed, and incubated at room temperature for 10 min. The mixture was added to cells and incubated for 24 h before cell lysis. In all cases, greater than 50% of cells were fluorescent. Transfected cells were washed twice with cold phosphate-buffered saline (PBS) and lysed with 1 mL of cold NP40 lysis buffer (50 mM Tris [pH 7.6], 150 mM NaCl, 0.5% NP40) supplemented with 0.1 mM phenylmethylsulfonyl fluoride (PMSF). Lysates were rotated at 4°C for a minimum of 1 h, then centrifuged at 12,000 rpm at 4°C for 10 min to remove insoluble material. The cleared lysates were incubated with rabbit anti-GFP antibody (A-6455; Invitrogen) with rotation at 4°C overnight before being conjugated to protein G Plus/Protein A agarose (Millipore Sigma) by rotation at 4°C for 3 h. Following the incubation, the agarose was washed three times with NP40 lysis buffer and resuspended in 25 µL of 2× final sample buffer (62.5 mM Tris [pH 6.8], 10% glycerol, 2% SDS, 0.01% bromophenol blue) for Western blot analysis.

### Assaying DUB activity in transfected cells

HEK293 cells seeded in 10-cm plates were transfected with plasmid constructs encoding GFP–strep–pUL36 [aa2–282] (pGS7900), and the mutant derivatives (pGS7901, pGS7902, and pGS7903), using Lipofectamine 3000 reagent (Thermo Fisher) according to the manufacturers protocol. Each 10-cm plate of HEK293 cells was incubated with a DNA–Lipofectamine 3000 mixture (20 µg of DNA, 60 µL of p3000, and 90 µL of Lipofectamine in 300 µL of DMEM per well) for 6 h followed by a media change and incubation for 48 h before cell lysis. In all cases, greater than 30% of cells were fluorescent. Transfected cells were washed twice with cold PBS and lysed with 1 mL of cold NP40 lysis buffer (50 mM Tris [pH 7.6], 150 mM NaCl, 0.5% NP40) supplemented with 0.1 mM PMSF. Lysates were rotated at 4°C for a minimum of 1 h, then centrifuged at 12,000 rpm for 30 min to remove insoluble material. GFP-Trap agarose beads (ChromoTek) were washed with GFP-Trap wash buffer (10 mM Tris [pH 7.6], 150 mM NaCl, 0.5 mM EDTA) and 30 µL of resuspended GFP-Trap agarose was added to each 1 mL of cleared lysate followed by rotation at 4°C for 1 h. The GFP-Trap agarose was washed three times with cold GFP-Trap wash buffer and was resuspended in 200 µL of HA-Ub-VME labeling buffer. HA-Ub-VME probe (4 µg from a 1 µg/µL stock in 50 mM Tris [pH 7.6] and 150 mM NaCl) was added to each sample, and the tubes were incubated at room temperature with rotation for 1 h. Following the incubation, the GFP-Trap agarose was washed three times with GFP-Trap wash buffer and resuspended in 25 µl of 2× final sample buffer (62.5 mM Tris [pH 6.8], 10% glycerol, 2% SDS, 0.01% bromophenol blue) for Western blot analysis.

### Western blot analysis

TEV protease-cleaved viral particles, HA-Ub-VME-labeled viral particles, HA-Ub-VME-labeled protein, and immunoprecipitated GFP–strep–pUL36 [aa2–282] were denatured in 2× final sample buffer supplemented with 10 mM DTT, boiled for 5 min, and separated on 4% to 20% SDS polyacrylamide gradient gels (Mini-PROTEAN TGX gel; Bio-Rad). Proteins were transferred to 0.45-µm-pore-size Immobilon-FL membranes (Millipore) and blocked in 5% (wt/vol) powdered milk in PBS (13.7 mM NaCl, 0.27 mM KCl, 1 mM Na_2_HPO_4_, 0.2 mM KH_2_PO_4_) for 80 min at room temperature. For detection of TEV protease-cleaved viral particles, PRV VP5 was detected using the mouse monoclonal anti-VP5 clone 3C10 (a gift from Lynn Enquist, Princeton University) at a dilution of 1:1,000, a mouse monoclonal GFP antibody (B2 SC-9996; Santa Cruz Biotechnology) was used to detect GFP-tagged pUL36 at a dilution of 1:1,000, and pUL36 was detected by a rabbit polyclonal antibody (pUL36 9K) that was generated by immunization with the PRV pUL36 peptide sequence HTVGGRPSRKFRPR at a dilution of 1:2,500. For detection of HA-Ub-VME-labeled viral particles, PRV pUL36 was detected by a rabbit polyclonal antibody (pUL36 9K) that was generated by immunization with the PRV pUL36 peptide sequence HTVGGRPSRKFRPR at a dilution of 1:2,500 and a mouse monoclonal HA antibody (G036; ABM) was used to detect HA-Ub-VME labeling at a dilution of 1:2,000. For detection of HA-Ub-VME-labeled purified protein, a rabbit polyclonal GFP antibody (A-6455; Invitrogen) was used to detect GFP-tagged pUL36 at a dilution of 1:4,000 and a mouse monoclonal HA antibody (G036; ABM) was used to detect HA-Ub-VME labeling at a dilution of 1:2,000. For detection of immunoprecipitated GFP–strep–pUL36 [aa2–282], a mouse monoclonal ubiquitin antibody (FK2, Enzo Life Sciences) was used to detect ubiquitin at a dilution of 1:1,000, the blot was then stripped, blocked, and a mouse monoclonal GFP antibody (B-2; Santa Cruz) was used to detect GFP-tagged pUL36 at a dilution of 1:1,000. Antibodies were prepared in 1% milk in PBST (PBS with 0.1% Tween 20) and incubated with membranes for 1 h to overnight at 4°C. Secondary antibodies were prepared in 1% milk–PBST and incubated with membranes for 60 min at room temperature in the dark. Detection was performed using a LI-COR Odyssey Fc imaging system with donkey anti-rabbit IRDye 680RD and goat anti-mouse IRDye 800CW secondary antibodies at a dilution of 1:10,000 for all cases except for immunoprecipitated GFP–strep–pUL36 where donkey anti-mouse IRDye 680RD was used to detect mouse anti-ubiquitin at a dilution of 1:5,000, and goat anti-mouse IRDye 800RD was used to detect mouse anti-GFP at a dilution of 1:5,000 (LI-COR Biosciences).

### Trigeminal ganglion invasion

Male CD-1 mice (6 weeks old; Charles River) were maintained for at least 2 weeks under a 12:12 L:D cycle, two to three mice per cage with food and water available *ad libitum*. Viral stocks were maintained frozen at −80°C and used immediately after being thawed while being maintained on ice. Intranasal application of PRV was administered to animals anesthetized by isoflurane (2.5%–5.0%) inhalation. Each animal received 5 µL of PRV (~1 × 10^5^ pfu) in each nostril,and the animals recovered almost immediately. Mice were maintained in a biosafety cabinet under a 12:12 L:D cycle. At 48 hpi, animals were deeply anesthetized and perfused transcardially with 0.9% saline followed by fixative consisting of 4% paraformaldehyde in phosphate buffer (0.1 M), pH 7.3, and the trigeminal ganglia (TG) were dissected. TG were post-fixed overnight in the same fixative containing 30% sucrose and serial sectioned at 40 µm with a CM1850 cryostat (Leica). Sections were mounted on microscope slides and imaged with a DM5500B fluorescence microscope (Leica) equipped with a C11440 Orca-flash 4.0 digital camera (Hamamatsu). The number of TG sensory neurons positive for red pUL25/mCherry fluorescence were counted on each section.

### Generation of predicted structural models

The PRV pUL36 residues 1–240 were used for structural prediction with the AlphaFold 2 algorithm through Colabfold v1.5.5 (68, 69). Amino acids 240–268 were excluded from the predictions because this region was not predicted with high confidence (PLDDT scores < 50), while residues 1–240 had PLDDT scores >70. The models generated with AlphaFold were visualized and aligned using PyMol software (The Pymol Molecular Graphics System, Version 3.0.5, Schrodinger, LLC). Five AlphaFold models for both WT and Mut5 were aligned with the crystal structure of the deubiquitinase domain of murine cytomegalovirus M48 (46), and the model with the lowest RMSD score was chosen for presentation and alignment of WT and Mut5.

## Data Availability

All underlying data are available upon request.

## SUPPLEMENTAL MATERIAL

Supplemental material jvi.01517-24-s0001.pdf Figures S1 to S7; Tables S1 to S3.

ASM does not own the copyrights to Supplemental Material that may be linked to, or accessed through, an article. The authors have granted ASM a non-exclusive, world-wide license to publish the Supplemental Material files. Please contact the corresponding author directly for reuse.
