# Supplementary material for: Tethered release of the pseudorabies virus deubiquitinase from the capsid promotes enzymatic activity
Source: J Virol. 2024 Dec 5;99(1):e01517-24. doi: 10.1128/jvi.01517-24 (PMC11784296; doi:10.1128/jvi.01517-24)
Supplement: Supplemental material — Figures S1 to S7; Tables S1 to S3. [file jvi.01517-24-s0001.pdf]

Figure S1

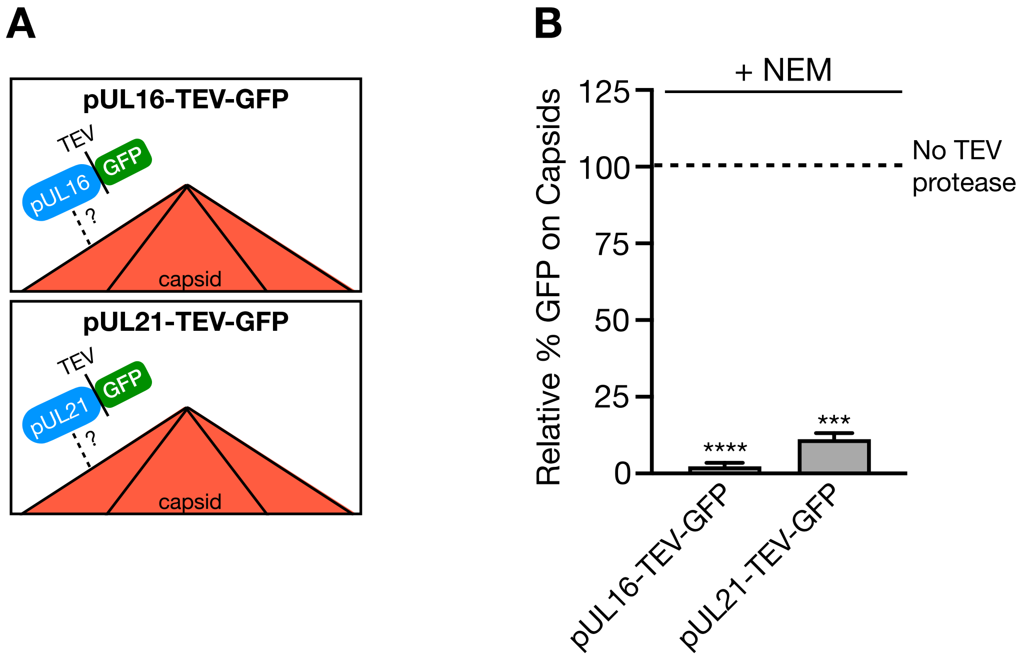

Fig S1. GFP release from pUL16 and pUL21 following NEM treatment and cleavage.

(A) Schematic of two PRV recombinants encoding TEV protease cleavage sites at the fusion junction between GFP and the C-terminus of pUL16 or pUL21. The question mark indicates a capsid association that has not been molecularly defined.

(B) GFP emissions from capsids processed by the NEM>TEV protease cleavage assay as illustrated in Fig 2A. Each virus encodes RFP-capsids, GFP fused to either pUL16 or pUL21, and a TEV protease cleavage site at the fusion junction. Values are normalized to the cognate "No TEV protease" control, which is set to 100% and represented by the dashed line (n = 3). Error bars indicate standard deviation (***, $P < 0.001$; ****, $P < 0.0001$ based on two-tailed unpaired t test with Welch correction).

Figure S2

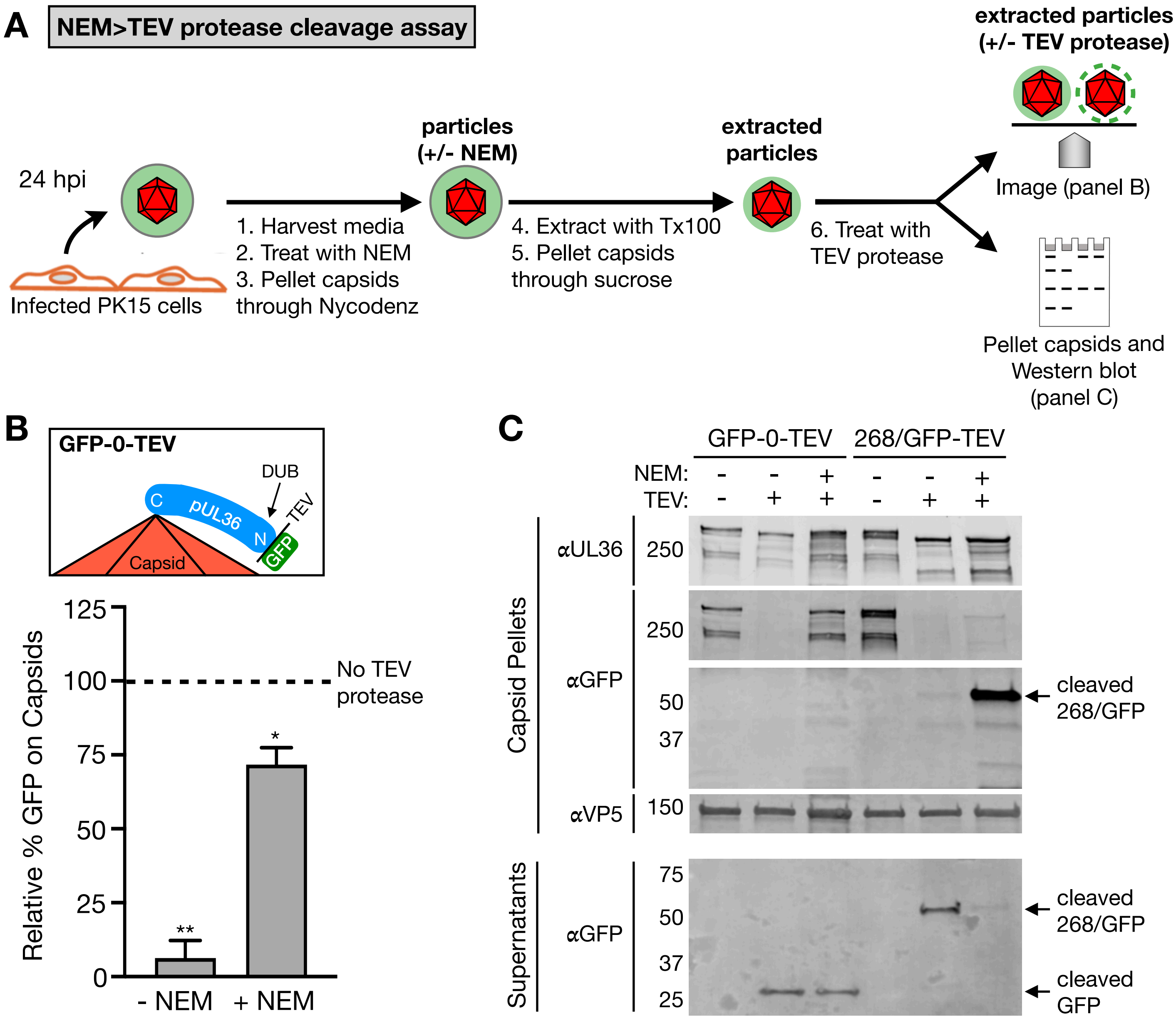

Fig S2. TEV protease cleavage of GFP-TEV fusions in pUL36.

(A) Experimental workflow of extracellular virus particle harvesting and processing in the NEM>TEV protease cleavage assay.

(B) Schematic of PRV recombinant encoding TEV protease cleavage site at the junction between GFP and pUL36 (aka, position zero). GFP emissions from PRV capsids processed in the NEM>TEV protease cleavage assay are shown below with values normalized to the cognate "No TEV protease" control, which is set to 100% and represented by the dashed line (*n* = 3). Error bars indicate standard deviation (*, $P < 0.05$; **, $P < 0.01$ based on two-tailed unpaired *t* test with Welch correction).

(C) TEV protease cleavage of indicated viral particles examined by Western blot. GFP-TEV fused to the pUL36 N-terminus (GFP-0-TEV) had poor cleavage following NEM treatment. The GFP-pUL36 design was abandoned because of this technical problem and the 268/GFP-TEV design was used instead.

Figure S3

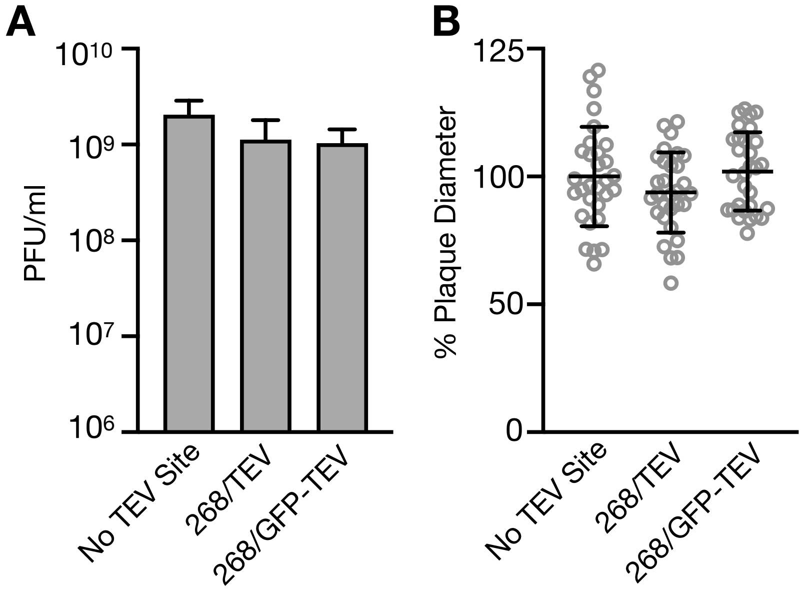

Fig S3. Propagation of PRV recombinants.

(A) Average viral titers determined by plaque assay (n=3).

(B) Plaque diameters presented as a percentage of the parental virus. Each virus encodes the RFP capsids and either unmodified pUL36 (No TEV Site), pUL36 with a TEV protease site inserted after aa268 (268/TEV), or pUL36 with GFP fused in-frame after aa268 followed by a TEV protease cleavage site (268/GFP-TEV). Error bars indicate standard deviation (no significance based on ordinary one-way ANOVA followed by Dunnett's multiple-comparison test).

Figure S4

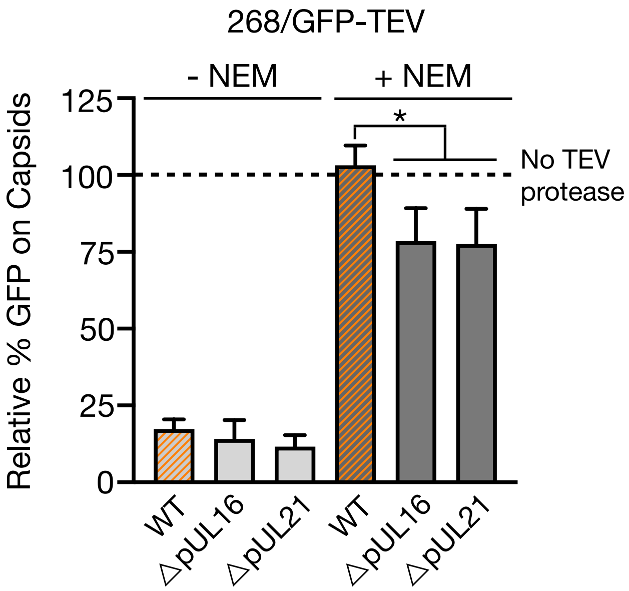

Fig S4. pUL36 N-terminal release and retention in the absence of pUL16 or pUL21.

GFP emissions from "268/GFP-TEV" capsids processed by the NEM>TEV protease cleavage assay. Each virus encodes RFP-capsids and GFP followed by a TEV protease cleavage site, as well as the indicated deletion. Values are normalized to the cognate "No TEV protease" control, which is set to 100% and represented by the dashed line ($n = 3$). WT data is duplicated from Fig 2C (highlighted in orange). Error bars indicate standard deviation (*, $P < 0.05$ based on ordinary one-way ANOVA followed by Dunnett's multiple-comparison test).

Figure S5

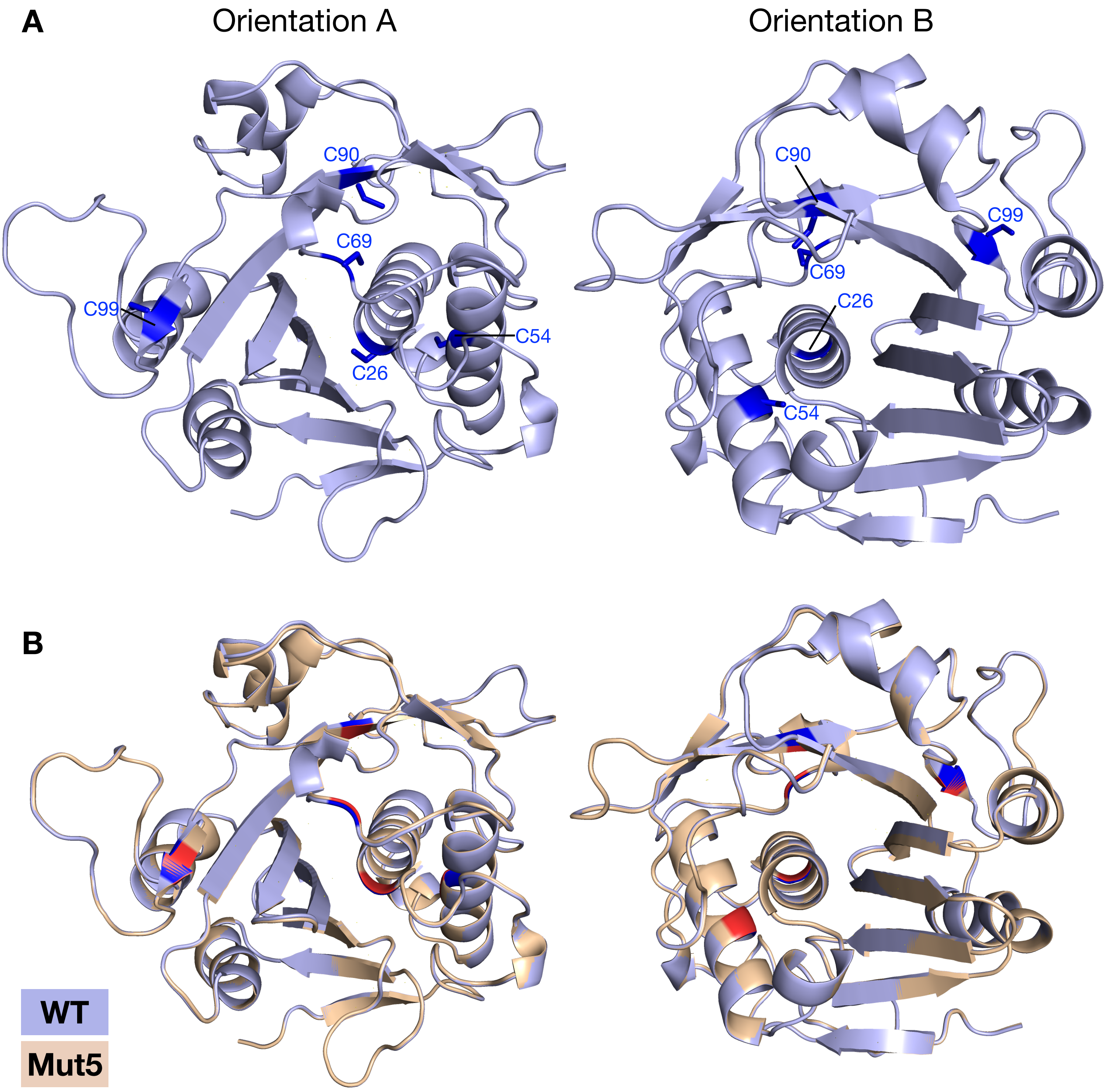

Fig S5. AlphaFold predicted structures of the PRV pUL36 N-terminal DUB domain.

(A) Predicted structure of pUL36 amino acids 1-240, highlighting the five cysteines (blue).

(B) Overlay of the predicted wild-type (WT) structure from panel A (lilac) and the Mut5 derivative (beige) with RMSD 0.101 Å. WT cysteines are in blue and Mut5 mutations are in red.

Figure S6

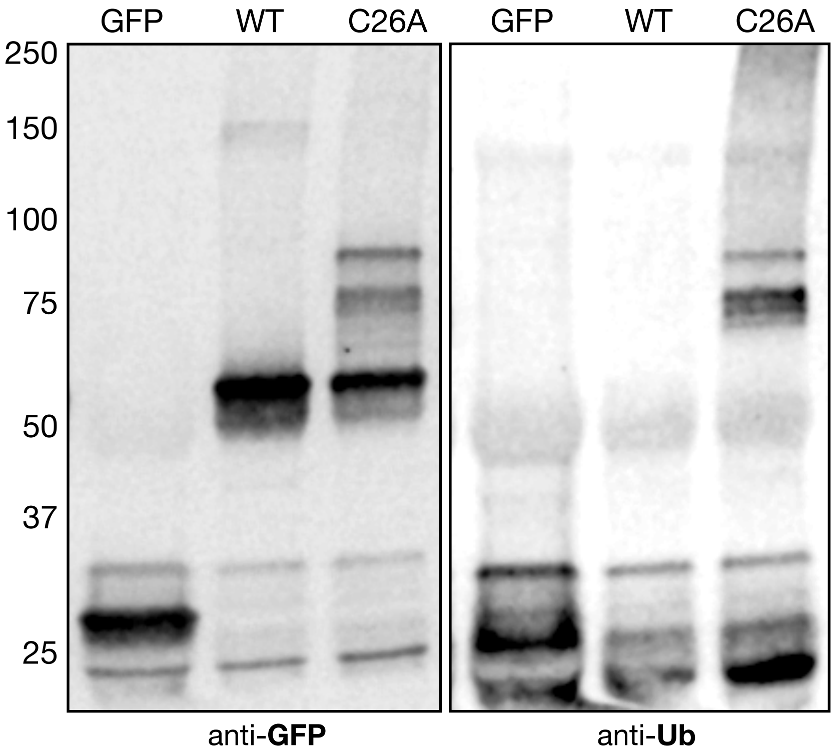

Fig S6. Ubiquitination of the pUL36 C26A mutant.

Transiently expressed GFP and GFP-pUL36 aa2-282 (WT and C26A mutant) were immunoprecipitated from HEK293 cell lysates and detected by Western blotting. An anti-GFP antibody was used to asses pUL36 abundance, and an FK2 antibody was used to detect ubiquitination.

Figure S7

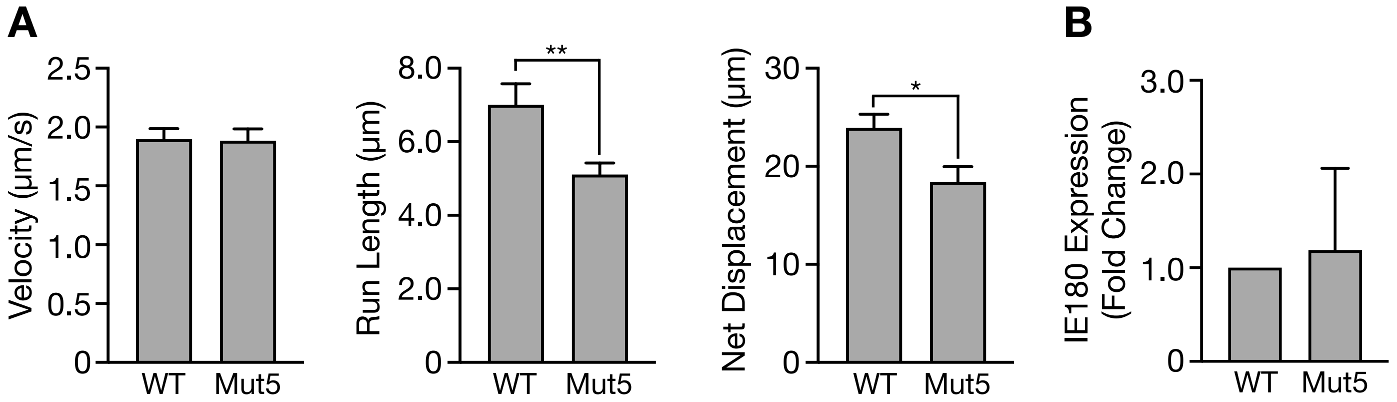

Fig S7. Retrograde axonal transport in primary sensory neurons and immediate early gene expression in epithelial cells infected with PRV WT and Mut5.

(A) RFP-capsid transport dynamics in axons was monitored for the first hour post infection, and average capsid velocities, run lengths, and net displacement were measured. Error bars indicate standard deviation (*, $P < 0.05$; **, $P < 0.01$ based on two-tailed unpaired *t* test).

(B) Expression of the PRV immediate early gene, IE180, was quantified by qRT-PCR and normalized to expression of the host S28 rRNA at 4 hpi with WT and Mut5. All values were plotted relative to levels observed during the WT infection. Four independent experiments were performed with each experimental replicate performed in triplicate. Values are expressed as mean ± standard deviation (not significant based on two-tailed unpaired *t* test with Welch correction).

Name	C26	C54	C69	C90	C99	DUB Release	DUB Activity
WT						82.6	1.00
C26A	A					97.5	0.00
C54S		S				78.4	0.82
C69S			S			86.8	1.45
$C90S_1$				S		80.1	0.93
$C90S_2$	rep	rep	rep	S		77.7	0.72
C99S					S	90.5	1.83
Mut2A	rep	S	S	rep	rep	32.9	0.02
Mut2B	rep	S	rep	S		16.6	-0.07
Mut2C	rep	S	rep	rep	S	45.1	0.19
$Mut2D_1$			S	S		25.5	-0.33
$Mut2D_2$	rep	rep	S	S		27.6	0.02
Mut2E			S		S	66.4	1.55
Mut2F				S	S	34.1	0.20
Mut3A	rep	S	S	S		6.9	0.03
Mut3B	rep	S	S	rep	S	15.5	-0.08
Mut4A	A	S	S	S		29	
Mut4B	A	S	S	rep	S	16.4	
Mut5	A	S	S	S	S	12.9	

Table S1. Summary of pUL36 cysteine mutants examined in this study.

Mutations of the five cysteine residues are detailed in the first 6 columns (A, mutation to alanine; S, mutation to serine; rep, repaired to cysteine). The two right columns summarize the results from Figs 4 and 5, respectively. The DUB Release column is derived from Fig 4 and indicates the amount of pUL36 N-terminus that released from capsids by TEV protease in the absence of NEM (values are 100 minus the normalized average percent remaining on capsids). Values in red are results that were significantly different from WT, indicating a lack of release of the pUL36 N-terminal fragment from capsids. The DUB Activity column is derived from Fig 5 and is a densitometry analysis of anti-HA signal divided by anti-pUL36 signal. Values are normalized to the WT and are subtracted for the C26A background value. Values in red are ≤ 20% of WT HA-Ub-VME labeling.

Table S2. Recombinant PRV used in this study

Strain	Label Name	Capsid tag	Tegument Modifications	pUL36 Mutations	Titer pfu/ml	Source
PRV-GS4284		pUL25/mCh			2.1×10^9	Ref 40
PRV-GS6370		pUL25/mCh	pUL16-GFP		7.3×10^8	This study
PRV-GS7550		pUL25/mCh	pUL16-TEV-GFP		1.7×10^8	This study
PRV-GS5692		pUL25/mCh	pUL21-GFP		7.8×10^8	This study
PRV-GS7551		pUL25/mCh	pUL21-TEV-GFP		1.8×10^8	This study
PRV-GS6063		pUL25/mCh	pUL47-GFP		3.8×10^8	Ref 9
PRV-GS6871		pUL25/mCh	pUL48-GFP		5.3×10^8	This study
PRV-GS6064		pUL25/mCh	pUL49-GFP		8.3×10^8	Ref 9
PRV-GS7111		pUL25/mCh	pUL36 GFP-0-TEV		1.9×10^9	This study
PRV-GS7474		pUL25/mCh	pUL36 268-TEV		1.1×10^9	This study
PRV-GS7613	WT	pUL25/mCh	pUL36 268/GFP-TEV		1.0×10^9	This study
PRV-GS7591		pUL25/mCh	pUL36 268/TEV-GFP-TEV		1.2×10^9	This study
PRV-GS7640		pUL25/mCh	pUL36 Δaa6-268 GFP-TEV	∆aa6-268	4.2×10^7	This study
PRV-GS7636	C26A	pUL25/mCh	pUL36 268/GFP-TEV	C26A	2.0×10^8	This study
PRV-GS7638	Mut5	pUL25/mCh	pUL36 268/GFP-TEV	C26A/C54S/C69S/C90S/C99S	3.5×10^7	This study
PRV-GS7764	Mut4A	pUL25/mCh	pUL36 268/GFP-TEV	C26A/C54S/C69S/C90S	5.0×10^8	This study
PRV-GS7762	Mut4B	pUL25/mCh	pUL36 268/GFP-TEV	C26A/C54S/C69S/C99S	1.5×10^8	This study
PRV-GS7805	Mut3A	pUL25/mCh	pUL36 268/GFP-TEV	C54S/C69S/C90S	7.0×10^8	This study
PRV-GS7800	Mut3B	pUL25/mCh	pUL36 268/GFP-TEV	C54S/C69S/C99S	1.2×10^9	This study
PRV-GS7829	Mut2A	pUL25/mCh	pUL36 268/GFP-TEV	C54S/C69S	1.5×10^8	This study
PRV-GS7823	Mut2B	pUL25/mCh	pUL36 268/GFP-TEV	C54S/C90S	6.5×10^8	This study

PRV-GS7824	Mut2C	pUL25/mCh	pUL36 268/GFP-TEV	C54S/C99S	9.3 x 10^{7}	This study
PRV-GS7946	$Mut2D_1$	pUL25/mCh	pUL36 268/GFP-TEV	C69S/C90S	4.5 x 10^{8}	This study
PRV-GS7827	$Mut2D_2$	pUL25/mCh	pUL36 268/GFP-TEV	C69S/C90S	6.0 x 10^{8}	This study
PRV-GS7919	Mut2E	pUL25/mCh	pUL36 268/GFP-TEV	C69S/C99S	1.4 x 10^{9}	This study
PRV-GS7918	Mut2F	pUL25/mCh	pUL36 268/GFP-TEV	C90S/C99S	1.5 x 10^{9}	This study
PRV-GS7853	C54S	pUL25/mCh	pUL36 268/GFP-TEV	C54S	1.2 x 10^{9}	This study
PRV-GS7854	C69S	pUL25/mCh	pUL36 268/GFP-TEV	C69S	3.5 x 10^{8}	This study
PRV-GS7855	$C90S_1$	pUL25/mCh	pUL36 268/GFP-TEV	C90S	2.4 x 10^{8}	This study
PRV-GS7937	$C90S_2$	pUL25/mCh	pUL36 268/GFP-TEV	C90S	5.3 x 10^{8}	This study
PRV-GS7856	C99S	pUL25/mCh	pUL36 268/GFP-TEV	C99S	3.1 x 10^{8}	This study
PRV-GS7631	ΔpUL16	pUL25/mCh	pUL36 268/GFP-TEV		2.7 x 10^{8}	This study
PRV-GS7632	ΔpUL21	pUL25/mCh	pUL36 268/GFP-TEV		2.0 x 10^{8}	This study

Table S3. Primers used to construct PRV recombinants unique to this study. Template homologies are in boldface.

Modification	PCR Template	Primer Sequence (5' - 3')
pUL16-GFP	pEP-EGFP-in	CCCCGCCCGCGCCATCCCCGAGCTAATAAACGATTAT**GTGAGCAAGGGCGAG**<br>GCCACAATACAAACGCAAGTACCCATTTTTTTCATTTCA**CTTGTACAGCTCGTC**
pUL16-TEV-GFP	pEP-EGFP-in	CCCCGCCCGCGCCATCCCCGAGCTAATAAACGATTATGAGAATTTGTACTTC CAAGGA**GTGAGCAAGGGCGAGGAG**<br>GCCACAATACAAACGCAAGTACCCATTTTTTTCATTTCA**CTTGTACAGCTCGTC**
pUL21-GFP	pEP-EGFP-in	GACCCGCGCCAACATCTCCATCGTCCTCAATAAAAACCGT**GTGAGCAAGGG CGAGGAG**<br>GACAGAGAACGGACGGGGGCGCGGACGTGGTGTTTGGTTA**CTTGTACAGC TCGTCCATGC**
pUL21-TEV-GFP	pEP-EGFP-in	GACCCGCGCCAACATCTCCATCGTCCTCAATAAAAACCGTGAGAATTTGTAC TTCCAAGGA**GTGAGCAAGGGCGAGGAG**<br>GACAGAGAACGGACGGGGGCGCGGACGTGGTGTTTGGTTA**CTTGTACAGC TCGTCCATGC**
pUL48-GFP	pEP-EGFP-in	CCTCATCCCCCGCGACGCGCTCAACCGGATGTTTGAGATG**GTGAGCAAGGG CGAGGAG**<br>AGGTGGTCGGGCGCCGCAGATCCGACCGCGCGGCGTCA**CTTGTACAGC TCGTCCATGC**
ΔpUL16	pEP-KanS2	CCGCGTGTAGCGGGGCGAGGGGGGCGGGCGCGCGCCATGTAATCCCCCGCC GCAAGA**AGGATGACGACGATAAGTAGGG**<br>TGAGCTCGGAGGCGACGTCGACGATCTTGCGGCGGGGGATTACATGGCGCGC GCCCG**CAACCAATTAACCAATTCTGATTAG**
ΔpUL21	pEP-KanS2	CGCCTCGCTCACCCATTTGCCGCGCCGCGTGTGCCCGCCGCCATGTAACCAA ACACCACG**AGGATGACGACGATAAGTAGGG**<br>TCGCGGACAGAGAACGGACGGGGGCGCGGACGTGGTGTTTGGTTACATGGCG GCGGGCAC**CAGTGTGATGGATATCTAGG**
pUL36 268/TEV	pEP-KanS2	GTCGCCCCCGTCCCCGCCGTCCCCGTCTCGGCACCCGGCGGCGAGAATTTG TACTTCCAAGGAGCACCGCTTCCGCCG**AGGATGACGACGATAAGTAGGG**<br>ATGCTCGGCCGCTTCTGGACCCGCGGCGACGGCGGAAGCGGTGCCCCTT GGAAGTACAAATTCTCGCCGCCGGGTGCCGA**CAGTGTGATGGATATCTAGG**
GFP-TEV-pUL36	pEP-KanS2	ACCGCCGCCGGGATCACTCTCGGCATGGACGAGCTGTACAAGGAGAATTTGTA CTTCCAAGGAACGGCCGACGCGGTG**AGGATGACGACGATAAGTAGGG**<br>GGGTCATACTGATTACGATAGCCGACGACCACCGCGTCGGCCGTCCCTTGGA AGTACAAATTCTCCTTGTACAGCTCGTC**CAGTGTGATGGATATCTAGG**
pUL36 268/GFP-TEV	pEP-EGFP-in	GTCGCCCCCGTCCCCGCCGTCCCCGTCTCGGCACCCGGCGGCGGAGGCG GT**GTGAGCAAGGGCGAGGAG**<br>GCTCGGCCGCTTCTGGACCCGCGGCGACGGCGGAAGCGGTGCCCCTTGGA AGTACAAATTCTCTCCTCCACC**CTTGTACAGCTCGTCCATGC**
pUL36 268/TEV-GFP-TEV	pEP-EGFP-in	GTCGCCCCCGTCCCCGCCGTCCCCGTCTCGGCACCCGGCGGCGAGAATTT GTACTTCCAAGGAGGAGGCGGT**GTGAGCAAGGGCGAGGAG**<br>GCTCGGCCGCTTCTGGACCCGCGGCGACGGCGGAAGCGGTGCCCCTTGGA AGTACAAATTCTCTCCTCCACC**CTTGTACAGCTCGTCCATGC**

pUL36 Δaa6-268 GFP-TEV	pEP-EGFP-in	CCCACGCGCGTGTGTTATTTCAGCCATGACGGCCGACGCGGGAGGCGGT**GTGAGCAAGGGCGAGGAG**<br>GCTCGGCCGCTTCTGGACCCGCGGCGACGGCGGAAGCGGTGCCCCTTGGAAGTACAAATTCTCTCCTCCACC**CTTGTACAGCTCGTCCATGC**
pUL36 C26A	pEP-KanS2	GGCTATCGTAATCAGTATGACCCCGACCTGGGGCCCGGGTCGGGCGTCTCAGCGCTGCGCTCCTCCCTC**AGGATGACGACGATAAGTAGGG**<br>CTCCACGCCGTTCGTGAAGACCAGGCGCAGGAAGGAGAGGGAGGAGCGCAGAGCTGAGACGCCCGACCC**CAACCAATTAACCAATTCTGATTAG**
pUL36 C54S	pEP-KanS2	ACGAACGGCGTGGAGGCCGCCCTGACGGCCGACGCCGTGGACGCGTCCCTGCGCGAGGGCCAG**AGGATGACGACGATAAGTAGGG**<br>GCACATGCCCCCGCCGGGCCCCGTCCACGCCTGGCCCTCGCGCAGGGACGCGTCCACGGCGTC**CAGTGTGATGGATATCTAGG**
pUL36 C69S	pEP-KanS2	TGCCTGCGCGAGGGCCAGGCGTGGACGGGGCCCGGCGGGGGCATGTCCGCCATCAGCGAGCTC**AGGATGACGACGATAAGTAGGG**<br>GCCCGCGCCCTCGTAGACGATCTTGTTCGGGAGCTCGCTGATGGCGGACATGCCCCCGCCGGG**CAGTGTGATGGATATCTAGG**
pUL36 C90S	pEP-KanS2	CCGAACAAGATCGTCTACGAGGGCGCGGGCGGCGCCGCGCTGCACTCCGTCTTCTCGAGGGTGCACGGCGAGT**AGGATGACGACGATAAGTAGGG**<br>AACCCTCGGCGGGCGGCGTGAAAAAGTTGCACTCGCCGTGCACCCTCGAGAAGACGGAGTGCAGCGCGGCGCC**CAGTGTGATGGATATCTAGG**
pUL36 C90S/C99S	pEP-KanS2	GGCGGCGCCGCGCTGCACTCCGTCTTCTCGCGGGTGCACGGCGAGTCCAACTTTTTCACGCCG**AGGATGACGACGATAAGTAGGG**<br>GATCTGCGTGCTCTGGAAACCCTCGGCGGGCGGCGTGAAAAAGTTGGACTCGCCGTGCACCCGC**AGTGTGATGGATATCTAGG**
pUL36 C26A>C repair	pEP-KanS2	CGTAATCAGTATGACCCCGACCTGGGGCCCGGGTCGGGCGTCTCGTGCCTGCGCTCCTCCCTC**AGGATGACGACGATAAGTAGGG**<br>GCCGTTCGTGAAGACCAGGCGCAGGAAGGAGAGGGAGGAGCGCAGGCACGAGACGCCCGACCC**CAGTGTGATGGATATCTAGG**
pUL36 C54S>C repair	pEP-KanS2	ACGAACGGCGTGGAGGCCGCCCTGACGGCCGACGCCGTGGACGCGTGCCTGCGCGAGGGCCAG**AGGATGACGACGATAAGTAGGG**<br>GCACATGCCCCCGCCGGGCCCCGTCCACGCCTGGCCCTCGCGCAGGCACGCGTCCACGGCGTCC**AGTGTGATGGATATCTAGG**
pUL36 C69S>C repair	pEP-KanS2	TGCCTGCGCGAGGGCCAGGCGTGGACGGGGCCCGGCGGGGGCATGTGCGCCATCAGCGAGCTC**AGGATGACGACGATAAGTAGGG**<br>GCCCGCGCCCTCGTAGACGATCTTGTTCGGGAGCTCGCTGATGGCGCACATGCCCCCGCCGGGC**AGTGTGATGGATATCTAGG**
pUL36 C99S>C repair	pEP-KanS2	GGCGGCGCCGCGCTGCACTGCGTCTTCTCGCGGGTGCACGGCGAGTGCAACTTTTTCACGCCG**AGGATGACGACGATAAGTAGGG**<br>GATCTGCGTGCTCTGGAAACCCTCGGCGGGCGGCGTGAAAAAGTTGCACTCGCCGTGCACCCGC**AGTGTGATGGATATCTAGG**
pUL36 C90S>C repair/C99S	pEP-KanS2	GGCGGCGCCGCGCTGCACTGCGTCTTCTCGCGGGTGCACGGCGAGTCCAACTTTTTCACGCCG**AGGATGACGACGATAAGTAGGG**<br>GATCTGCGTGCTCTGGAAACCCTCGGCGGGCGGCGTGAAAAAGTTGGACTCGCCGTGCACCCG**CAGTGTGATGGATATCTAGG**
